# Reduced mitochondrial calcium uptake in macrophages is a major driver of inflammaging

**DOI:** 10.1038/s43587-023-00436-8

**Published:** 2023-06-05

**Authors:** Philip V. Seegren, Logan R. Harper, Taylor K. Downs, Xiao-Yu Zhao, Shivapriya B. Viswanathan, Marta E. Stremska, Rachel J. Olson, Joel Kennedy, Sarah E. Ewald, Pankaj Kumar, Bimal N. Desai

**Affiliations:** 1 Pharmacology Department, University of Virginia School of Medicine, Charlottesville, VA USA; 2 Carter Immunology Center, University of Virginia School of Medicine, Charlottesville, VA USA; 3 Microbiology, Immunology, and Cancer Biology Department, University of Virginia School of Medicine, Charlottesville, VA USA; 4 Biochemistry and Molecular Genetics Department, University of Virginia School of Medicine, Charlottesville, VA USA; 5 University of Virginia, Bioinformatics Core, Charlottesville, VA USA

**Keywords:** Inflammation, Mitochondria, Ageing, Ion channel signalling

## Abstract

Mitochondrial dysfunction is linked to age-associated inflammation or inflammaging, but underlying mechanisms are not understood. Analyses of 700 human blood transcriptomes revealed clear signs of age-associated low-grade inflammation. Among changes in mitochondrial components, we found that the expression of mitochondrial calcium uniporter (*MCU*) and its regulatory subunit *MICU1*, genes central to mitochondrial Ca^2+^ (mCa^2+^) signaling, correlated inversely with age. Indeed, mCa^2+^ uptake capacity of mouse macrophages decreased significantly with age. We show that in both human and mouse macrophages, reduced mCa^2+^ uptake amplifies cytosolic Ca^2+^ oscillations and potentiates downstream nuclear factor kappa B activation, which is central to inflammation. Our findings pinpoint the mitochondrial calcium uniporter complex as a keystone molecular apparatus that links age-related changes in mitochondrial physiology to systemic macrophage-mediated age-associated inflammation. The findings raise the exciting possibility that restoring mCa^2+^ uptake capacity in tissue-resident macrophages may decrease inflammaging of specific organs and alleviate age-associated conditions such as neurodegenerative and cardiometabolic diseases.

## Abstract (web summary)

Seegren et al. demonstrate that the mitochondrial calcium uptake complex is a key molecular apparatus that links age-related changes in mitochondrial physiology to systemic macrophage-mediated age-associated inflammation.

## Main

Inflammation is widely recognized as a key driver of aging^1,2^. An age-associated low-grade, chronic inflammatory state promotes tissue damage and hence this process is referred to as inflammaging. The etiology of inflammaging is not understood but it is thought to involve an increase in the baseline inflammatory output by immune cells, as evident from higher cytokine levels and other inflammatory markers in the blood of aged humans^3–6^. Inflammatory stimuli can originate from multiple sources: pathogens, resident microbiomes, tissue damage-associated inflammatory signals, and even spontaneous production of inflammatory molecules by senescent cells^7–9^. Myeloid cells of the immune system, such as macrophages and neutrophils, are central players in inflammation and may contribute to inflammaging.

Macrophages reside in every organ system and act as sentinel cells monitoring their environment for infection or injury^10–12^. The inflammatory gene expression in macrophages is a highly regulated process with multiple checkpoints. The nuclear factor kappa B (NF-κB) family of dimeric transcription factors have an evolutionarily conserved and central role in inflammatory gene expression^13,14^. Many studies have pointed to the salience of NF-κB to inflammaging^15–18^. Analysis of age-related changes in gene expression in human and mouse tissues identified the NF-κB pathway as the most strongly associated transcriptional pathway to aging^15^. The secretion of high levels of pro-inflammatory cytokines in two different mouse models of accelerated aging was also found to be dependent on abnormal NF-κB activaton^17^. These studies suggest that a lowered threshold of NF-κB activation underlies inflammaging, but how this transpires is not understood. Many positive and negative signaling elements control the activation of NF-κB^13^. Among these regulatory checkpoints, the nuclear translocation and transcriptional activity of NF-κB is also controlled by cytosolic Ca^2+^ (cCa^2+^) signaling^19–22^.

Ca^2+^ is a ubiquitous and essential second messenger in cell biology^23^. Elevations in cCa^2+^ trigger an influx of Ca^2+^ into the mitochondrial matrix through the mitochondrial calcium uniporter (MCU), a Ca^2+^-selective ion channel that resides in the mitochondrial inner membrane^24–30^. The mitochondrial outer membrane is porous to ions, but the inner membrane has a resting membrane potential between −160 mV and −200 mV, relative to the cytosol^24,31^. MICU1 (refs. ^32,33^) and MICU2 (refs. ^34^), the EF-hand containing Ca^2+^-sensitive regulatory subunits of MCU interact directly with MCU in the intermembrane space. Structural studies support the view that MCU–MICU1–MICU2 interactions are configured to have a switch-like sensitivity to [Ca^2+^], enabling rapid mCa^2+^ uptake when cytosolic [Ca^2+^] increases beyond the resting range of ~10–100 nM^35–38^. Because the mitochondrial matrix contains many metabolic enzymes that are regulated by Ca^2+^, the mCa^2+^ signaling within the matrix has a profound effect on mitochondrial physiology and metabolism^29,39,40^. The cells of the vertebrate immune system use Ca^2+^ signaling for an immediate-early response to antigenic and inflammatory stimuli—cCa^2+^ elevations regulate the activation of both the innate and adaptive immune cells^41^. Recently, we revealed that mCa^2+^ signaling functions as an electrometabolic switch to fuel macrophage-mediated phagosomal killing^42^. The process involves a fast two-step Ca^2+^ relay to meet the bioenergetic demands of phagosomal killing. Additionally, recent reports have supported a role for the MCU and mCa^2+^ in macrophage polarization^43,44^, host defense^42,45^ and tissue homeostasis^46–48^. mCa^2+^ is thus emerging as a central node for innate immunity and inflammatory responses. Here we report a surprising discovery that mCa^2+^ uptake capacity of macrophages decreases progressively with age, and this is a major driver of inflammaging.

## Results

### Human blood transcriptomes reveal signs of age-associated low-grade inflammation

**Fig. 1:**
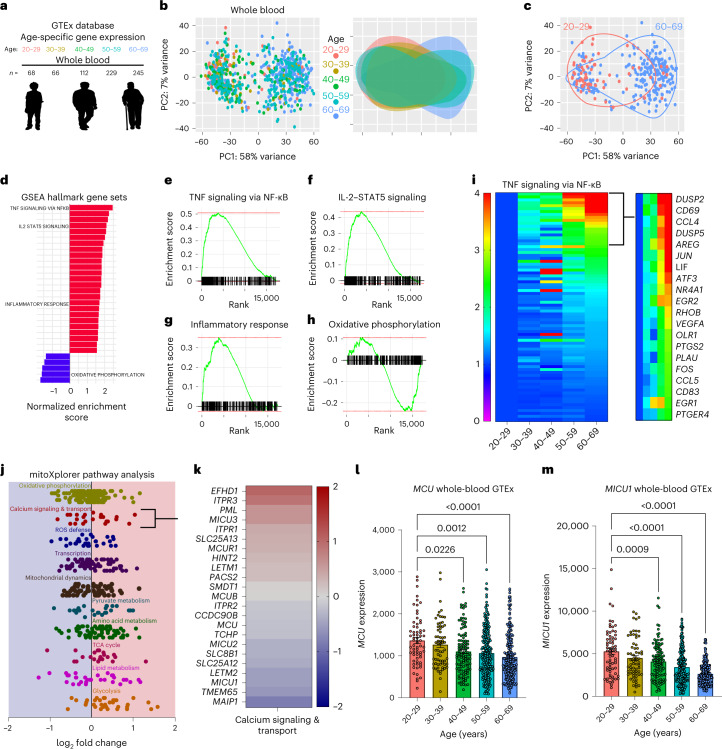
Age-related changes in whole-blood gene expression are associated with increased inflammatory gene transcription and decreased expression of genes encoding mitochondrial Ca^2+^ transport. **a**, GTEx database mined for tissue-specific gene expression across five indicated age groups; note the color coding for the age groups. **b**, Left, PCA of whole-blood gene expression from every sample, color coded according to the age groups. Right, the same data were used and color-coded clusters from each age group were overlaid. Note the variance in gene expression from different age groups. **c**, PCA plots from **b** were used to show only samples from the youngest and oldest age groups analyzed. **d**, Hallmark GSEA based on differential gene expression between the oldest (60–69 years) and youngest (20–29 years) datasets. Pathways were ranked by *P* value and plotted on the *x* axis by the normalized enrichment scores. **e**, GSEA of GSEA hallmark pathway, TNF signaling via NF-κB, based on differential gene expression of oldest (60–69 years) versus youngest (20–29 years) GTEx samples. Enrichment scores are plotted on the *y* axis and genes ranked in the ordered dataset are plotted on the *x* axis. **f**, GSEA of the IL-2–STAT5 GSEA hallmark pathway. **g**, GSEA of inflammatory response GSEA hallmark pathway. **h**, GSEA of the oxidative phosphorylation GSEA hallmark pathway. **i**, Heat map of expression levels of genes associated with the TNF–NF-κB pathway. Expression values were calculated as a fold change from the 20–29-year age group. **j**, mitoXplorer Pathway analysis based on DSeq2 analysis of oldest (60–69 years) versus youngest (20–29 years) GTEx samples. **k**, Individual genes in the calcium signaling and transport pathway were identified from mitoXplorer pathway analysis based on DSeq2 analysis of oldest (60–69 years) versus youngest (20–29 years) GTEx samples. Fold change was determined as a relative change in 60–69-year compared to 20–29-year GTEX samples. **l**, *MCU* gene counts for each sample in the GTEx database sorted by age. Error bars reflect the s.e.m.; *P* values were calculated using ordinary one-way analysis of variance (ANOVA). **m**, *MICU1* gene counts for each sample in GTEx database sorted by age. Error bars reflect the s.e.m.; *P* values were calculated using ordinary one-way ANOVA. Source data

**Extended Data Fig. 1:**
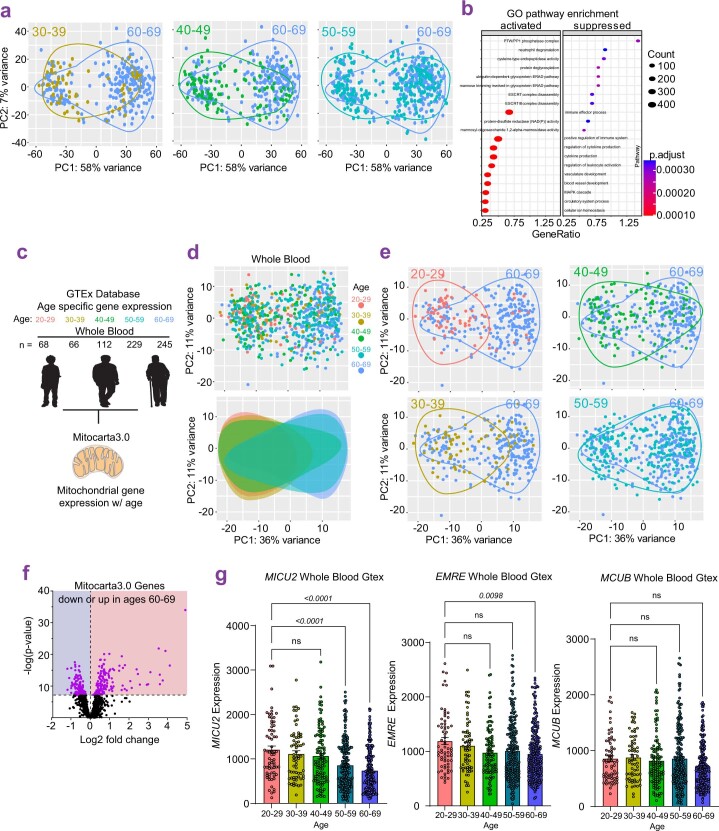
GTEx analysis of human whole blood transcriptomes. **a**. Variance in gene expression in whole blood of different age groups based on Principal Component analysis on GTEx samples. **b**. GO pathway enrichment analysis based on DSeq2 from GTEx samples. Differential gene expression is plotted for ages 60–69 vs 20–29. Significance determined by Dotplot function (clusterProfiler R package^1^). **c**. Pipeline used for the analysis of genes encoding for mitochondria-localized proteins (mito-genes). **d**. Principal component analysis on mito-genes from GTEx samples. Variance in gene expression is shown for individual age groups. **e**. Variance in gene expression shown for age 20–29 vs 60–69, 30–39 vs 60–69, 40–49 vs 60–69, and 50–59 vs 60–69. **f**. Volcano plot of mito-genes expression levels in old (60–69y) vs young (20–29y) samples. Data normalization, dispersion estimates, and model fitting (negative binomial) were carried out with the DESeq function (DESeq2 R package). Wald statistics were used for the significance tests. **g**. *MICU2, EMRE, and MCUB* gene counts for each sample in GTEx database sorted by age. Error bars reflect SEM; *p-values* were calculated using one-way ANOVA. Source data

To gain insights into inflammaging, we mined the publicly available Genotype-Tissue Expression (GTEx) database (https://gtexportal.org/)^49^ for tissue-specific gene expression across five different human age groups (Fig. 1a). Because mature red blood cells are anucleated and do not contain any appreciable amounts of mRNA, RNA sequencing (RNA-seq) of whole blood is a reasonable surrogate of combined gene expression in the white blood cells and platelets. Expression profile data were obtained for different tissues, binned into age groups, and then subjected to differential gene expression analysis using DESeq2 R package. Principle-component analysis (PCA) plots, from the five different age groups, revealed clear age-associated clustering (Fig. 1b). The variance in overall gene expression was greatest when we compared the youngest population (age 20–29 years) with the oldest population (age 60–69 years; Fig. 1c), but the variance in overall gene expression was the least when we compared the second-oldest population (age 50–59 years) to the oldest population (age 60–69 years; Extended Data Fig. 1a). These data substantiate the view that gene expression in the blood changes significantly with age. To derive further insights and to distill testable hypotheses for the etiology of inflammaging, we focused our analysis on differences between the youngest (age 20–29 years) and oldest (age 60–69 years) samples. Gene-set enrichment analysis (GSEA) hallmark and GO pathway analyses of differentially expressed genes showed that the genes associated with inflammatory responses were upregulated in the older population (Fig. 1d and Extended Data Fig. 1b). Additionally, we observed a significant decrease in the gene expression related to oxidative phosphorylation, a mitochondrial process, in the older populations. Enrichment scores and gene ranks of inflammatory genes (Fig. 1e–g) and oxidative phosphorylation-associated genes (Fig. 1h) suggest a significant dysregulation in these pathways in the aged population. As shown in the heat map (Fig. 1i), genes associated with the NF-κB pathway were upregulated in the blood cells of older humans. Overall, these gene expression analyses show clear signs of age-associated chronic low-grade inflammation in the whole blood of human samples.

### Gene expression of mitochondrial Ca^2+^ uptake machinery correlates inversely with age

**Extended Data Fig. 2:**
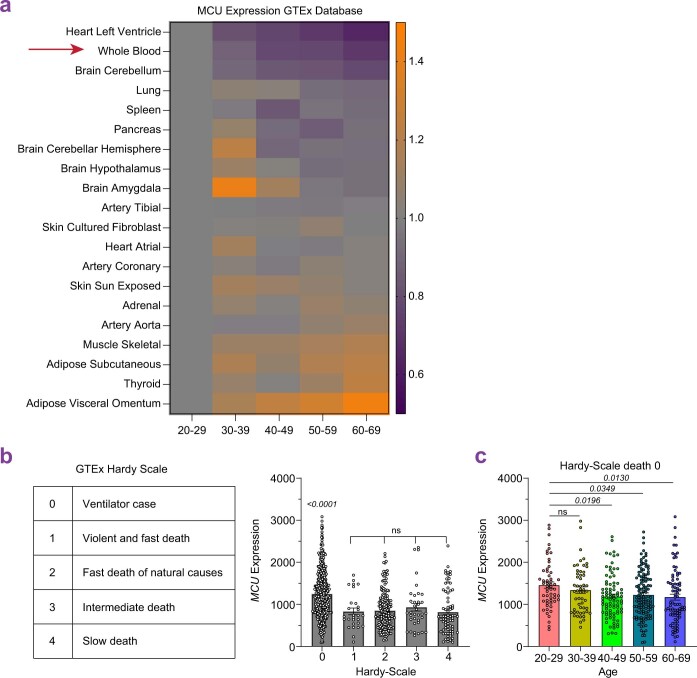
Age-associated changes in *MCU* expression in different human tissues. **a**. Heat map for fold change gene reads of *MCU* across different tissues. **b**. *MCU* gene counts for each sample in GTEx database sorted by 4-point Hardy-Scale. Error bars reflect SEM; *p-values* were calculated using one-way ANOVA. **c**. *MCU* gene counts for each sample in GTEx database sorted by age for all death circumstance 0 on 4-point Hardy-Scale. Error bars reflect SEM; *p-values* were calculated using one-way ANOVA. Source data

Both inflammation and mitochondrial dysfunction are hallmarks of aging^1^, and we wondered if there was a relationship pertinent to inflammaging. For the analysis of age-related changes in mitochondrial function, we used mitoXplorer, an analysis and visualization tool specialized for genes associated with mitochondrial functions (mito-genes)^50^. In accordance with previous observations, we observed significant age-related changes in the expression of mito-genes (Extended Data Fig. 1c–f). We noted a decrease in the mito-genes associated with oxidative phosphorylation, calcium signaling and reactive oxygen species defense (Fig. 1j). The mito-genes associated with mitochondrial transcription, mitochondrial dynamics, pyruvate metabolism and amino acid metabolism were expressed at similar levels. The lipid metabolism, tricarboxylic acid (TCA) cycle and glycolysis genes were expressed at higher levels in the aged population. Because Ca^2+^ signaling has a direct impact on inflammatory signaling in immune cells, we considered genes involved in mCa^2+^ signaling and found decreased expression of *MCU*, *MICU1* and *MICU2* (Fig. 1k). Moreover, the decrease in *MCU* and *MICU1* expression was strongly associated with age, decreasing progressively as humans age (Fig. 1l,m). These observations suggested an age-associated dysregulation in mCa^2+^ uptake in the blood-borne immune cells. This transcriptional dysregulation was observed for *MCU*, *MICU1* and *MICU2* but the gene expression of *EMRE* (*SMDT1*) and the dominant-negative regulator *MCUB* showed no significant change with age (Fig. 1l,m and Extended Data Fig. 1g). We wondered if such an age-related decrease in *MCU* is found in all human tissues. We checked different tissues in the age-stratified GTEx data we had mined and found that the age-associated decrease in *MCU* gene expression was only seen in a few tissues—heart, whole blood and cerebellum (Extended Data Fig. 2a). The vast majority of tissues did not show decreased *MCU* expression, and some tissues, skeletal muscle, adipose tissue and thyroid showed the opposite trend—*MCU* expression in these tissues increased with age. The participant death parameters are reported in the GTEx database on a four-point Hardy Scale (Extended Data Fig. 2b). We assessed *MCU* expression across the reported Hardy Scale and found that the *MCU* expression was higher in the most abundant category (death 0), compared to other death categories (Extended Data Fig. 2b). The participants in death category 0 were on a ventilator before their death. When we analyzed the whole-blood samples of participants binned in this category (death 0), we still observed an age-dependent decrease in *MCU* (Extended Data Fig. 2c). Together, these results suggest that mCa^2+^ uptake capacity changes with age in some tissues, and likely contributes to age-related changes in the physiology of these tissues. From the standpoint of age-associated inflammation, the analyses put a spotlight on the key finding that in the blood, expression of *MCU* and *MICU1* decrease progressively with age.

### Reduced mitochondrial Ca^2+^ uptake in macrophages derived from old mice

**Fig. 2:**
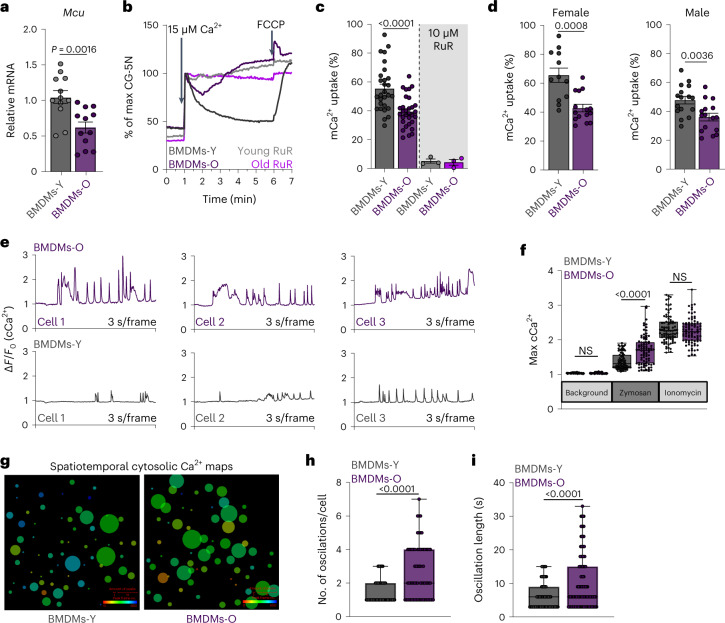
Macrophages generated from aged mice display decreased mitochondrial Ca^2+^ uptake. **a**, *Mcu* expression in BMDMs. *N* = 12 biological replicates, from four mice. Error bars reflect the s.e.m.; *P* = 0.0016 according to Welch’s *t*-test, two-tailed. **b**, Representative traces of mCa^2+^ uptake. **c**, Quantification of **b**. *N* = 28 biological replicates. Error bars reflect the s.e.m.; *P* values were calculated using ordinary one-way ANOVA. RuR, Ruthenium red. **d**, mCa^2+^ uptake data shown in **c**—segregated by sex. Error bars reflect the s.e.m.; *P* values were calculated using Welch’s *t*-test, two-tailed. **e**, Representative cCa^2+^ oscillations. **f**, Maximum cCa^2+^. *N* = 88 cells, three independent experiments. Whiskers represent the minimum to maximum values for each dataset. The box represents the 75th and 25th percentiles. The line is the median; *P* < 0.0001 according to one-way ANOVA. **g**, CALIMA spatiotemporal Ca^2+^ dynamics. **h**, Number of oscillations in individual cells. *N* = 88 cells, three independent experiments. Whiskers represent the minimum to maximum values for each dataset. The box represents the 75th and 25th percentiles. Line is at the median; *P* < 0.0001 according to Welch’s *t*-test, two-tailed. **i**, Oscillation length in individual cells. *N* = 88 cells, three independent experiments. Whiskers represent the minimum to maximum values for each dataset. The box represents the 75th and 25th percentiles. Line is at the median; *P* < 0.0001 according to Welch’s *t*-test, two-tailed. NS, not significant. Source data

**Extended Data Fig. 3:**
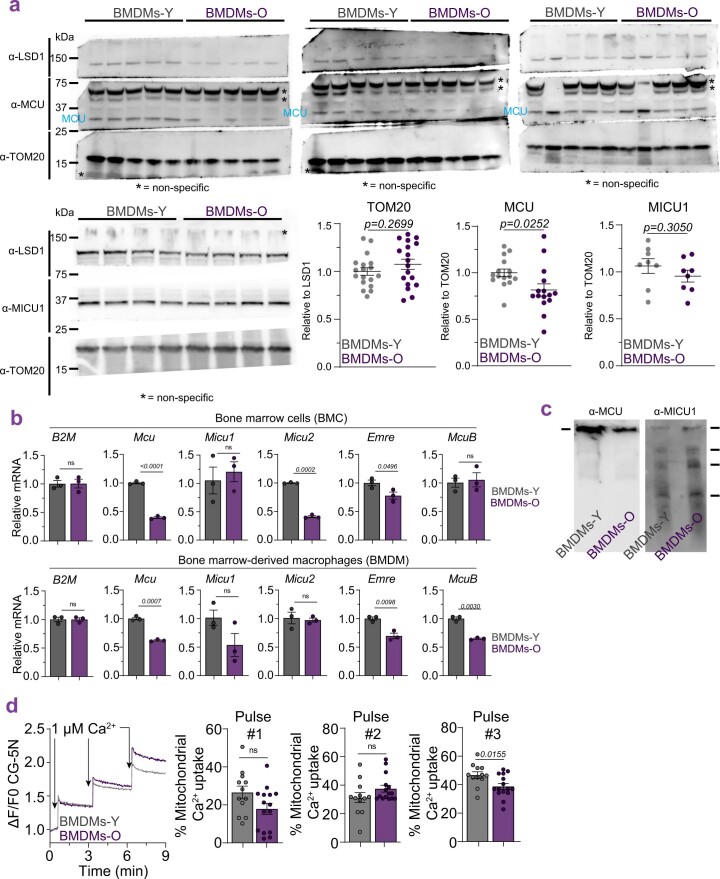
Expression of MCU components in BMDMs. **a**. Western blot analysis of MCU, TOM20*, and* MICU1 protein. N = 5–15 mice, error bars reflect SEM; *p-value* determined by Welch’s t-test, two-tailed. **b**. Quantitative-PCR of *Mcu* and its regulatory subunits. N = 3 mice. Error bars reflect SEM; *p-value* determined by Welch’s t-test, two-tailed. **c**. Resolution of MCU complex in non-reducing conditions and immunoblotting for MCU and MICU1, N = 1 mouse. **d**. Pulsed mitochondrial Ca^2+^ uptake. N = 15 biological replicates. Source data

**Extended Data Fig. 4:**
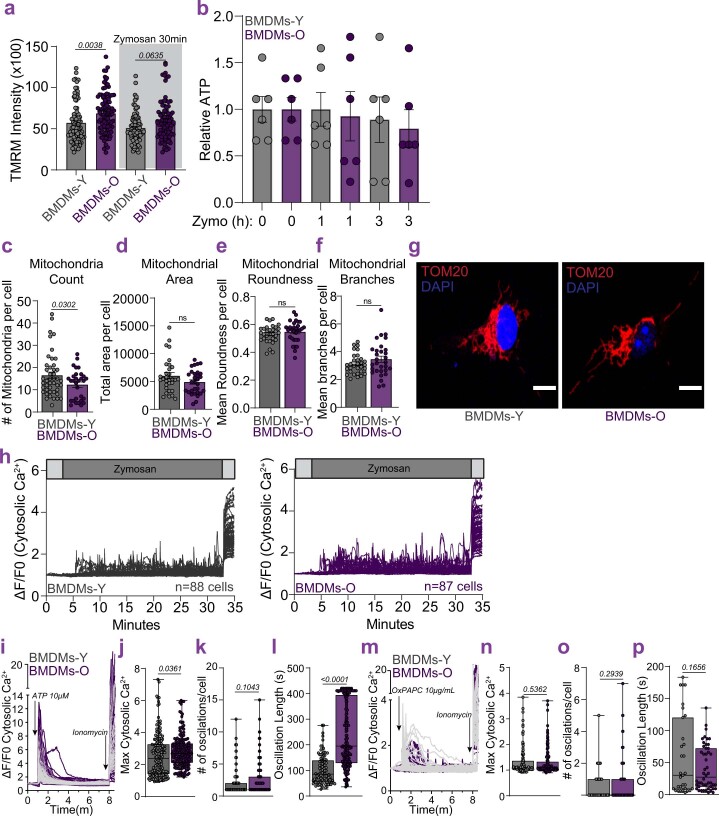
Analysis of mitochondria and Ca^2+^ responses in BMDMs. **a**. Mitochondrial membrane potential. N = 90 cells, 3 independent experiments. Error bars reflect SEM; *p-value* determined by one-way ANOVA. **b**. Normalized luminescent measurements of ATP. N = 6 biological repeats. Error bars reflect SEM; *p-value* determined by one-way ANOVA. **c**. Mitochondrial counts. N = 30–45 cells, 3 independent experiments. Error bars reflect SEM; *p-value* significance according to Welch’s t-test, two-tailed. **d**. Mitochondrial area. N = 30–45 cells, 3 independent experiments. Error bars reflect SEM; not statistically significant according to Welch’s t-test, two-tailed. **e**. Mitochondrial roundness. N = 30–45 cells, 3 independent experiments. Error bars reflect SEM; not statistically significant according to Welch’s t-test, two-tailed. **f**. Mitochondrial branches. N = 30–45 cells, 3 independent experiments. Error bars reflect SEM; not statistically significant according to Welch’s t-test, two-tailed. **g**. Representative images of *BMDMs-Y* and *BMDMs-O* immunostained for TOM20. Scale bar at 10 µm. **h**. Cytosolic Ca^2+^ Oscillations in *BMDMs-Y* (n = 88 cells) and *BMDMs-O* (n = 87 cells). **i**. Cytosolic Ca^2+^ Oscillations with ATP. **j**. Maximum cytosolic Ca^2+^. N = 112 cells, 2 independent experiments. Whiskers represent the min to max values for each data set. Box represents 75^th^ and 25^th^ percentile. Line is at the median; *p-value* according to Welch’s t-test, two-tailed. **k**. Number of oscillations in individual cells. N = 112 cells, 2 independent experiments. Whiskers represent the min to max values for each data set. Box represents 75^th^ and 25^th^ percentile. Line is at the median; *p<value* according to Welch’s t-test, two-tailed. **l**. Oscillation length in individual cells. N = 112 cells, 2 independent experiments. Whiskers represent the min to max values for each data set. Box represents 75^th^ and 25^th^ percentile. Line is at the median; *p<0.0001* according to Welch’s t-test, two-tailed. **m**. Cytosolic Ca^2+^ Oscillations with OxPAPC. **n**. Maximum cytosolic Ca^2+^. N = 104 cells, 2 independent experiments Whiskers represent the min to max values for each data set. Box represents 75^th^ and 25^th^ percentile. Line is at the median; *p-value* according to Welch’s t-test, two-tailed. **o**. Number of oscillations in individual cells. N = 104 cells, 2 independent experiments. Whiskers represent the min to max values for each data set. Box represents 75^th^ and 25^th^ percentile. Line is at the median; *p<value* according to Welch’s t-test, two-tailed. **p**. Oscillation length in individual cells. N = 104 cells, 2 independent experiments. Whiskers represent the min to max values for each data set. Line is at the median; *p<0.0001* according to Welch’s t-test, two-tailed. Source data

The most abundant cell types in the human blood are myeloid cells, which are composed mainly of neutrophils and monocytes. Both of these cell types are short-lived in the blood but play a crucial role in inflammation. We reasoned that monocytes are more important for chronic low-grade inflammation because they can differentiate into macrophages and thereby sustain low-grade inflammation and inflammatory cascades over a relatively long period of time. Furthermore, all tissues and organs contain specialized resident macrophages, which are central to local inflammation and homeostasis. We also know that mCa^2+^ uptake plays an important role in macrophage-mediated fungal killing^42^. Considering these aspects, we focused our investigation on how mCa^2+^ signaling might affect macrophage-mediated inflammation. First, we confirmed that the age-associated decrease in *Mcu* expression was recapitulated in mouse bone marrow-derived macrophages from older mice (BMDMs-O) when compared to those from the young mice (BMDMs-Y; Fig. 2a). Importantly, the reduced gene expression resulted in decreased MCU protein levels (Extended Data Fig. 3a). MICU1 protein levels were unchanged (Extended Data Fig. 3a). We wondered if this transcriptional defect was a result of macrophage differentiation ex vivo or intrinsic to bone marrow progenitors. We measured the gene expression of *Mcu* and its regulatory subunits in undifferentiated bone marrow cells (BMCs) and bone marrow-derived macrophages (BMDMs) isolated from young (15–25 weeks) and old (80–90 weeks) mice (Extended Data Fig. 3b). In the old BMCs, we found a significant decrease in the expression of *Mcu*, *Micu2* and *Emre*. In the BMDMs derived from old BMCs, we found a significant decrease in the gene expression of *Mcu*, *Emre* and *Mcub*. These results indicate that the bone marrow progenitors undergo substantial changes in the expression of MCU complex components, and especially in the expression of *Mcu*. Changes in regulatory subunit composition and expression can affect mCa^2+^ uptake capacity^51^. We reasoned that gross changes in the stoichiometry of the MCU complex would affect its protein mobility when resolved on a non-reducing gel. However, the mobility was identical in BMDMs-O and BMDMs-Y (Extended Data Fig. 3c). Stripping the membrane and immunoblotting for MICU1 showed comparable levels of MICU1 at the same mobility at MCU, although we found MICU1 in other complexes as well (Extended Data Fig. 3c). Next, we tested the most obvious hypothesis that macrophages exhibit an age-dependent decrease in their mCa^2+^ uptake. The basic technical design of this assay is to add Ca^2+^ to macrophages permeabilized with digitonin, and as the mitochondria take up the added Ca^2+^, its loss from the bath solution is reported by the reduction in the fluorescence of calcium green-5N, a small-molecule Ca^2+^ indicator in the bath solution. We show that BMDMs derived from the young mice exhibited robust mCa^2+^ uptake, but this process was significantly impaired in the BMDMs-O. The representative traces are shown in Fig. 2b and a quantification of the percentage of the added Ca^2+^ taken up by the mitochondria is shown in Fig. 2c. The addition of the mitochondrial uncoupler FCCP stops the Ca^2+^ uptake and even reverses it (Fig. 2b), indicating that the mCa^2+^ uptake is driven by the membrane potential of the mitochondrial inner membrane. Similarly, Ruthenium red (10 μM), a known blocker of MCU^24,31^, abrogates mCa^2+^ uptake, showing that the process is largely dependent on MCU. This age-associated reduction in mCa^2+^ uptake was found in both females and males (Fig. 2d). When we pulsed a much lower dose of Ca^2+^ (1 µM), the mCa^2+^ uptake in BMDMs-O was comparable for the first two pulses but started to lag behind BMDMs-Y after that (Extended Data Fig. 3d), consistent with impaired mCa^2+^ uptake. To determine if defects in mCa^2+^ uptake were a result of decreased mitochondrial membrane potential, we measured mitochondrial membrane potential in BMDMs-O and BMDMs-Y with TMRM, at baseline and after zymosan stimulation (Extended Data Fig. 4a). Surprisingly, BMDMs-O showed a modest hyperpolarization compared to BMDMs-Y suggesting the defect in mCa^2+^ uptake is independent of resting membrane potential (Extended Data Fig. 4a). We also measured ATP levels in BMDMs-Y and BMDMs-O but found no significant differences in ATP levels (Extended Data Fig. 4b). Besides an evaluation of mCa^2+^ uptake, we also quantified mitochondrial numbers and morphology. We immunostained for TOM20 and then applied an automated image processing software to quantify mitochondrial numbers and morphology of confocal images^52^. Comparing BMDMs-Y and BMDMs-O in this manner, we found a modest reduction in mitochondrial numbers but no significant differences in mitochondrial area, roundness and branches (Extended Data Fig. 4c–g). Overall, the results show conclusively that the macrophages in old mice have a significant defect in mCa^2+^ uptake, and this is attributable, at least in part, to a substantial decrease in MCU protein levels and to modest changes in mitochondrial numbers. Next, we focused on understanding the functional implications of this age-associated defect in mCa^2+^ uptake in macrophages.

### Amplified cytosolic Ca^2+^ oscillations in BMDMs-O responding to zymosan

We hypothesized that a reduction in mCa^2+^ uptake would disrupt cCa^2+^ signaling, which is crucial for inflammatory signaling. We challenged BMDMs derived from young and old mice with zymosan, a fungal glucan wherein the glucose monomers are polymerized through β-1,3 glycosidic bonds. Zymosan is a potent stimulator of both Toll-like receptor 2 (TLR2) and dectin-1 (CLEC7A) receptors on myeloid cells. The downstream activation of phospholipase C-gamma (PLC-γ) elicits a robust store-operated Ca^2+^ entry (SOCE), which involves an initial release of endoplasmic reticulum (ER)-resident Ca^2+^ stores, followed by more sustained entry of extracellular Ca^2+^ through the ORAI channels. Inflammatory gene expression mediated by multiple transcription factors, especially NF-κB, is highly sensitive to cCa^2+^ oscillations^19,20^. In response to zymosan, the amplitudes of the cCa^2+^ oscillations in BMDMs-O were significantly elevated compared to BMDMs-Y; typical and representative traces from cells are shown (Fig. 2e and Extended Data Fig. 4h). A statistical comparison of the maximum Ca^2+^ elevations achieved in each cell also shows that the BMDMs-O achieved significantly higher amplitudes (Fig. 2f). In these experiments, ionomycin, a Ca^2+^ ionophore, was used as a positive control demonstrating that both cell populations were loaded equivalently with the Ca^2+^ dye (FURA-2AM) and were thus capable of reporting higher and equivalent levels of Ca^2+^. The overlaid traces of cCa^2+^ from all cells are also shown (Extended Data Fig. 4h). The spatial distribution of these oscillations across the imaging field (containing many cells) is also informative but not captured by such a traditional display of Ca^2+^ oscillations. For a deeper analysis of this aspect of Ca^2+^ dynamics, we used CALIMA, an image analysis software specially designed to measure spatiotemporal aspects of Ca^2+^ oscillations^53^. The spatial distribution of Ca^2+^ oscillations in representative image fields is shown (Fig. 2g) with the origin of each circle at the cellular location and the diameter proportional to the number of spikes originating from that location. The color spectrum of the circles denotes the time at which that location first reported a spike. For instance, in each field, the location of the reddish-brown circle reported a Ca^2+^ spike earlier than the circles colored green and so on. These spatial maps clearly show that BMDMs-O exhibit a significantly higher number of Ca^2+^ oscillations for each cell and they also start spiking sooner than BMDMs-Y. These are quantified (Fig. 2h), and the differences in oscillatory lengths are also shown (Fig. 2i). These data establish that Ca^2+^ elevations are amplified in the BMDMs-O during inflammatory signaling. Our analysis on Ca^2+^ dynamics in response to fungal pathogens is highly relevant to chronic low-grade inflammation attributed to dysregulated microbiome and ‘leaky gut’ observed in older populations^54^. However, we were curious if this may also pertain to other mechanisms of chronic low-grade inflammation. Two additional sources of low-grade inflammation in older populations are ATP release from dying cells and oxidative stress. We subjected BMDMs-O to analyses of cCa^2+^ dynamics in response to ATP (Extended Data Fig. 4i–l) and oxidized PAPC (OxPAPC; Extended Data Fig. 4m–p). Although the nature of Ca^2+^ response to ATP is different from that to zymosan, the BMDMs-O showed dysregulated cCa^2+^ responses with an increase in maximum amplitudes (Extended Data Fig. 4j) and oscillation lengths (Extended Data Fig. 4l). This was not the case for OxPAPC-triggered Ca^2+^ responses—we did not find any significant differences between BMDMs-Y and BMDMs-O. This dichotomy suggests that mCa^2+^ uptake doesn’t play a major role in OxPAPC-triggered Ca^2+^ elevations.

### Inconsistent changes in gene expression of store-operated Ca^2+^ entry components

**Fig. 3:**
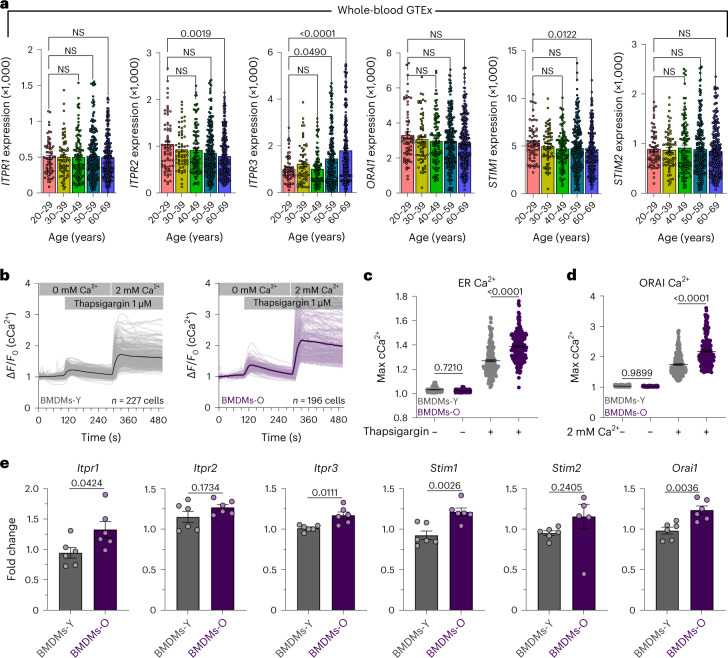
Store-operated Ca^2+^ entry responses in macrophages generated from aged mice. **a**, *ITPR1*, *ITPR2*, *ITPR3*, *ORAI1*, *STIM1* and *STIM2* gene counts for each sample in whole-blood GTEx database sorted by age. Error bars reflect the s.e.m.; *P* values were calculated using ordinary one-way ANOVA. **b**, Representative cCa^2+^ oscillations in BMDMs-Y and BMDMs-O. Bold lines indicated the mean of individual traces. **c**, Quantification of ER Ca^2+^. *N* = 150 cells, three independent experiments. Error bars represent the s.e.m.; *P* values were determined by one-way ANOVA. **d**, Quantification of ORAI Ca^2+^. *N* = 150 cells, three independent experiments. Error bars represent the s.e.m.; *P* value determined by one-way ANOVA. **e**, Fold change expression at baseline for SOCE genes. *N* = 6 biological replicates, two independent experiments. Error bars represent the s.e.m.; *P* value determined by Welch’s *t*-test, two-tailed. Source data

Recent reports in mice have suggested ER Ca^2+^ channels as important drivers of cellular aging and senescence^55^. Since mCa^2+^ uptake is thought to occur in close juxtaposition to ER Ca^2+^ release^56^, we analyzed the whole-blood GTEx transcriptome for age-associated changes in SOCE machinery. We observed inconsistent changes in the expression of SOCE components. There was an upregulation in *ITPR3* but downregulation of *ITPR1* (Fig. 3a). Additionally, we observed a modest decrease in *STIM1* expression but no effect of age on *ORAI1* or *STIM2* expression (Fig. 3a). These observations do not support a clear role for changes in SOCE machinery. We also assessed composite SOCE responses in BMDMs-O and BMDMs-Y (Fig. 3b). In these experiments, macrophages were loaded with Fura-2-AM and placed in a 0 mM Ca^2+^ bath and stimulated with thapsigargin, a sarcoplasmic reticulum Ca^2+^ ATPase (SERCA) pump inhibitor, to visualize ER Ca^2+^ release (first elevation in cCa^2+^). This is followed by the addition of 2 mM extracellular Ca^2+^ to observe the ORAI-dependent Ca^2+^ influx (second elevation; Fig. 3b). Loss of mCa^2+^ uptake is predicted to increase both the first cCa^2+^ elevation caused by the release of Ca^2+^ from the ER and the second elevation caused by the entry of extracellular Ca^2+^ into the cytosol. Quantification of the ER Ca^2+^ release and ORAI Ca^2+^ entry revealed increased SOCE responses in BMDMs-O (Fig. 3c,d). Although reduced mCa^2+^ uptake is a major component of amplified SOCE, additional factors like increased release from the ER cannot be ruled out (Fig. 3e). Regardless of the mechanistic intricacies, amplified cCa^2+^ responses are a hallmark of BMDMs-O.

### *Mcu*^−/−^ BMDMs-Y recapitulate the amplified cytosolic Ca^2+^ signals seen in BMDMs-O

**Extended Data Fig. 5:**
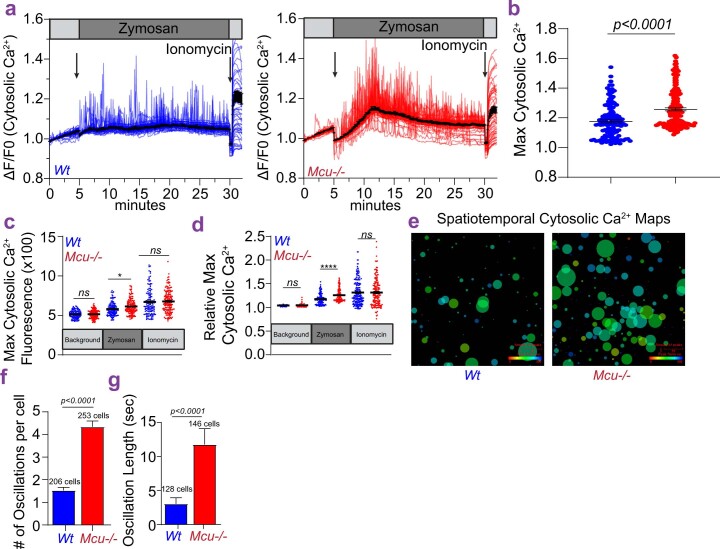
cCa^2+^ dynamics in *Mcu−/−* BMDMs-Y. **a**. Cytosolic Ca^2+^ Oscillations in *wt* and *Mcu−/−* BMDMs. **b**. Maximum cytosolic Ca^2+^. N = 83 cells, 2 independent experiments. Error bars represent SEM; *p<0.0001* according to Welch’s t-test, two-tailed. **c**. Maximum cytosolic Ca^2+^. N = 83 cells, 2 independent experiments. Error bars represent SEM; p<0.0001(****), p≤0.001(***), p≤0.01(**), p≤0.05(*) determined by the Brown-Forsythe and Welch ANOVA test. **d**. Maximum cytosolic Ca^2+^. N = 83 cells, 2 independent experiments. Error bars represent SEM of two independent experiments; p<0.0001(****), p≤0.001(***), p≤0.01(**), p≤0.05(*) determined by the Brown-Forsythe and Welch ANOVA test. **e**. CALIMA maps depicting spatiotemporal aspects of cytosolic Ca^2+^ elevations. **f**. Average number of oscillations per cell, seen during zymosan stimulation. N = 83 cells, 2 independent experiments Error bars represent SEM; *p<0.0001* according to Welch’s t-test, two-tailed. **g**. Mean of oscillation lengths during zymosan stimulation. N = 83 cells, 2 independent experiments Error bars represent SEM; *p<0.0001* according to Welch’s t-test, two-tailed. Source data

**Extended Data Fig. 6:**
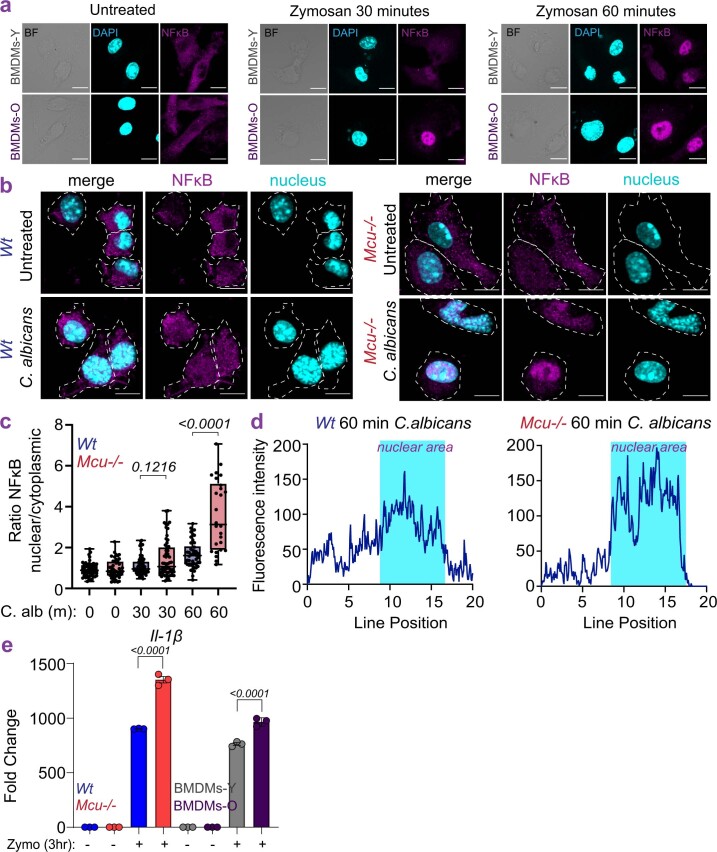
Analysis of NFkB translocation in response to *C. albicans*. **a**. Representative images for data shown in Fig. 3g shown as single channels. **b**. Representative images from *wt* and *Mcu−/−* BMDMs, untreated or stimulated with *C. albicans*, for 30 or 60 minutes and immunostained for NFκB p65 subunit and nuclei (DAPI). The outline of cells was determined by bright field images. **c**. Nuclear to cytoplasmic ratios of the fluorescent intensity of NFκB for images shown in *panel b*. N = 30–72 cells, 2 independent experiments. Whiskers represent the min to max values for each data set. Box represents 75^th^ and 25^th^ percentile. Line is at the median; *p-value* according to one-way ANOVA. **d**. Representative analysis of fluorescent intensity of p65 staining along a line drawn across the cytoplasm and nucleus, as determined by DAPI staining (shaded blue). **e**. Zymosan-induced IL-1b expression (qPCR) in indicated BMDMs. N = 3 biological replicates. Error bars represent SEM, *p-value* calculated by one-way ANOVA. Source data

To establish that this effect on cCa^2+^ signaling is because of reduced mCa^2+^ uptake, we show that the effect can be robustly recapitulated in BMDMs derived from young mice (age range, 15–25 weeks) by simply deleting *Mcu*. As shown (Extended Data Fig. 5a), *Mcu*^*−/−*^ BMDMs, derived from *Mcu*^fl^*(Cx3cr1-cre)* mice^42^ show a robust amplification of cCa^2+^ oscillations in response to zymosan. The maximum Ca^2+^ levels achieved by individual cells are shown in Extended Data Fig. 5b. Appropriate controls for long-term Ca^2+^ imaging is shown in Extended Data Figs. 5c and 6d. The spatiotemporal analysis using CALIMA is shown in Extended Data Fig. 5e–g. This analysis shows that, as seen in old BMDMs, the *Mcu*^*−/−*^ BMDMs exhibit many more oscillations for each cell, and the length of the oscillations is also increased. Together, these data establish that the increased cCa^2+^ signaling in the old BMDMs is a direct consequence of the reduced mCa^2+^ uptake.

### BMDMs-O and *Mcu*^−/−^ BMDMs-Y show signatures of senescence

**Fig. 4:**
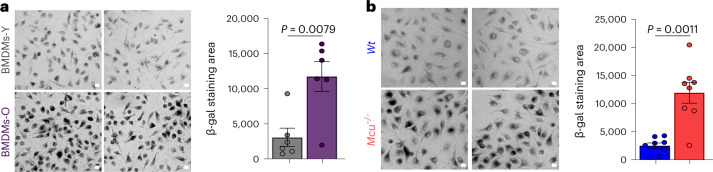
BMDMs-O and young *Mcu*^*−/−*^ macrophages show signatures of senescence. **a**, SA-β-gal activity. Scale bar, 10 µm. *N* = 6 biological replicates. **b**, SA-β-gal activity. Scale bar, 10 µm. *N* = 8 biological replicates. In **a** and **b**, error bars reflect the s.e.m., and *P* values were calculated using Welch’s *t*-test, two-tailed. Source data

Aging is commonly associated with the presence of senescent cells and a long-standing hallmark of senescent cells is senescence-associated β-galactosidase (SA-β-gal) activity attributed to the lysosomal β-galactosidase in mammals^57,58^. To determine if BMDMs-O exhibited signs of cellular senescence, we compared SA-β-gal staining in BMDMs-O and BMDMs-Y (Fig. 4a). In this method, cells were stained with X-gal and imaged for the presence of blue precipitates formed by the activity of SA-β-gal. These precipitates can be observed under a bright-field light microscope and appear as dark aggregates. By measuring hundreds of cells, we found that BMDMs-O showed significantly increased SA-β-gal staining (Fig. 4a). We reasoned that if the loss of mCa^2+^ uptake contributes to this senescent signature, we would observe SA-β-gal activity in *Mcu*^*−/−*^ macrophages from young mice. Indeed, *Mcu*^*−/−*^ macrophages from young mice had robust SA-β-gal activity (Fig. 4b). These findings bolstered the hypothesis that loss of mCa^2+^ uptake in macrophages renders them hyper-inflammatory and as facilitators of inflammaging.

### Hyper-inflammatory responses in both wild-type BMDMs-O and *Mcu*^−/−^ BMDMs-Y

**Fig. 5:**
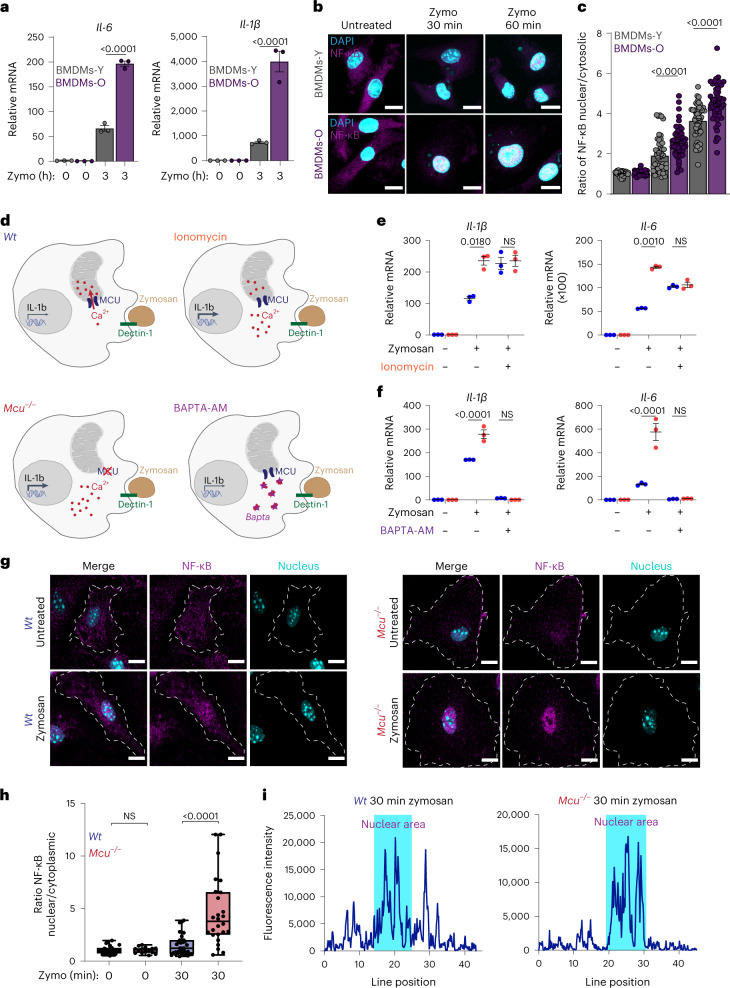
Mitochondrial Ca^2+^ uptake buffers cytosolic Ca^2+^ to control inflammatory output in macrophages. **a**, IL-6 and IL-1β mRNA expression from indicated BMDMs. Representative experiment, from two independent experiments. Error bars represent the s.e.m.; *P* < 0.0001 according to one-way ANOVA. **b**, Representative images from indicated BMDMs stimulated with zymosan for 30 and 60 min. Magenta shows immunostaining of NF-κB p65 subunit; cyan shows DAPI staining of nuclei. **c**, Nuclear to cytoplasmic ratios of the fluorescence intensity of NF-κB. *N* = 45 cells, three independent experiments. Bars reflect means of ratios; *P* values were determined by one-way ANOVA. **d**, Model depicting how mCa^2+^ uptake affects cCa^2+^ and inflammatory gene expression in response to zymosan. **e**, Effect of ionomycin on IL-1β and IL-6 mRNA expression in WT and *Mcu*^−/−^ macrophages. Representative experiment, from two independent experiments. Error bars represent the s.e.m.; *P* values were determined by two-way ANOVA. **f**, Effect of BAPTA-AM on IL-1β and IL-6 expression in WT and *Mcu*^−/−^ macrophages. Representative experiment, from two independent experiments. Error bars represent the s.e.m.; *P* value determined by two-way ANOVA. **g**, Representative images from WT and *Mcu*^−/−^ macrophages, untreated or stimulated with zymosan for 30 min and immunostained for NF-κB p65 subunit and nuclei (DAPI). **h**, Nuclear to cytoplasmic ratios of the fluorescence intensity of NF-κB. *N* = 25 cells, from three independent experiments. Whiskers represent the minimum to maximum values for each dataset. The box represents the 75th and 25th percentiles. The line is the median; *P* < 0.0001 according to one-way ANOVA. **i**, Representative analysis of fluorescence intensity of p65 staining along a line drawn across the cytoplasm and nucleus (DAPI staining), which is shaded blue. Source data

**Extended Data Fig. 7:**
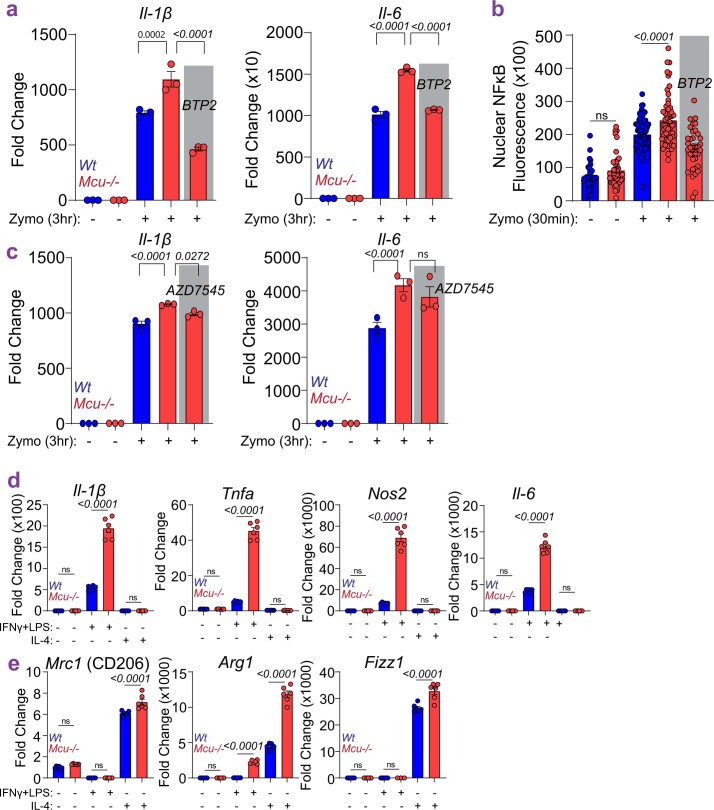
Effects of BTP2 and AZD7545 on inflammatory gene expression and analysis of macrophage activation. **a**. Fold change expression of *IL-1*β and *IL-6*. N = 3 biological replicates. Error bars represent SEM; *p-value* determined by one-way ANOVA. **b**. Nuclear NFκB. N = 36–50 cells, 3 independent experiments. Error bars represent SEM; *p-value* determined by one-way ANOVA. **c**. Fold change expression of *IL-1*β and *IL-6*. N = 3 biological replicates. Error bars represent SEM; *p-value* determined by one-way ANOVA. **d**. Assessment of M1 polarization in *wt* and *Mcu−/−* BMDMs. N = 6 biological replicates. Error bars represent SEM; *p-value* determined by one-way ANOVA. **e**. Assessment of M2 polarization in *wt* and *Mcu−/−* BMDMs. N = 6 biological replicates. Error bars represent SEM; *p-value* determined by one-way ANOVA. Source data

We hypothesized that the abnormally increased cCa^2+^ signaling in BMDMs-O would result in increased inflammatory output. Indeed, in response to zymosan, BMDMs-O expressed higher levels of pro-inflammatory cytokines interleukin (IL)-6 and IL-1β when compared to BMDMs-Y (Fig. 5a). NF-κB plays a crucial role in the transcription of these pro-inflammatory genes, and its activation is highly sensitive to Ca^2+^ signaling^19,59^. We measured NF-κB translocation (p65) in BMDMs-Y and BMDMs-O stimulated with zymosan and found that, in accordance with our model, the translocation of NF-κB was significantly enhanced in the BMDMs-O (Fig. 5b and Extended Data Fig. 6a). The quantification of the ratio of nuclear to cytoplasmic NF-κB shows that significantly more NF-κB translocated to the nuclei of BMDMs-O (Fig. 5c). These data show that macrophages from the older mice are hyper-inflammatory in response to zymosan. Consistently, we found that *Mcu*^*−/−*^ macrophages from young mice also exhibit a hyper-inflammatory response to zymosan stimulation, establishing a model wherein reduction in mCa^2+^ uptake increases inflammatory output through amplified cCa^2+^ signaling (Fig. 5d). Predictably, the *Mcu*^−/−^ BMDMs-Y showed significantly increased expression of both IL-1β and IL-6 (Fig. 5e). Note that when ionomycin was added to artificially increase cCa^2+^, even wild-type (WT) BMDMs-Y increased the expression of IL-1β, highlighting the sensitivity of the macrophage inflammatory response to cCa^2+^. A similar effect was observed for IL-6, but there was a key difference—while ionomycin increased the expression of IL-6 in WT cells, it also decreased the expression of IL-6 in *Mcu*^−/−^ BMDMs. A possible reason for this is that unlike the oscillatory effects caused by reduced mCa^2+^ uptake, ionomycin causes a global and sustained elevation of Ca^2+^. In *Mcu*^−/−^ BMDMs, this elevation is unbuffered by mCa^2+^ uptake, and this may inhibit other regulatory elements of IL-6 transcription. Nevertheless, the upregulation of both IL-1β and IL-6 is highly dependent on Ca^2+^ signaling. BAPTA-AM, a cell permeable, high-affinity Ca^2+^ chelator that prevents the elevation of cCa^2+^ during zymosan-triggered inflammatory signaling completely abrogated the expression of IL-1β and IL-6 (Fig. 5f). NF-κB translocation was also found to be enhanced in *Mcu*^−/−^ BMDMs-Y (Fig. 5g). Quantification of the nuclear/cytoplasmic ratio revealed an increase in NF-κB activation in *Mcu*^−/−^ BMDMs-Y (Fig. 5h). Representative line intensity plots across the nucleus and cytosol are shown (Fig. 5i). The increased activation of NF-κB in *Mcu*^−/−^ macrophages was also seen when they were stimulated with the fungal pathogen *Candida albicans* (Extended Data Fig. 6b), but the translocation kinetics were slower in comparison to zymosan stimulation (Extended Data Fig. 6c). Representative line intensity plots are shown (Extended Data Fig. 6d). The inflammatory output, as measured by *IL1B* gene expression, of *Mcu*^−/−^ BMDMs was higher than that of BMDMs-O because mCa^2+^ uptake was almost completely abrogated in *Mcu*^−/−^ BMDMs, while it was reduced in BMDMs-O (Extended Data Fig. 6e). During the macrophage response to zymosan, the influx of extracellular Ca^2+^ into the cytosol depends predominantly on Orai channels. Predictably, treating the *Mcu*^−/−^ BMDMs with an inhibitor of Orai, the main conduit of SOCE, greatly blunted the gene expression of both IL-1β and IL-6 (Extended Data Fig. 7a). BTP2 also blunted the nuclear translocation of NF-κB (Extended Data Fig. 7b). However, besides buffering cCa^2+^ elevations, mCa^2+^ uptake also regulates mitochondrial metabolism, primarily by regulating the activity of pyruvate dehydrogenase (PDH) complex and the TCA cycle^60^. In principle, the changes in mitochondrial metabolism in the *Mcu*^−/−^ BMDMs could exert an added effect on macrophage inflammatory output. Although this possibility cannot be completely ruled out, the following evidence suggests that changes in mitochondrial metabolism play a minimal role in the immediate regulation of inflammatory outputs in *Mcu*^−/−^ BMDMs. The influx of Ca^2+^ into the mitochondrial matrix regulates the TCA cycle through the activation of PDH complex. The PDH complex is activated through dephosphorylation by the Ca^2+^-activated PDH phosphatase (PDP)^61^ and abrogation of mCa^2+^ uptake prevents the dephosphorylation (and activation) of PDH. We reasoned that treating the cells with AZD7545, an inhibitor of the PDH kinase, would counter the lack of PDP activity and restore PDH activity in a Ca^2+^-independent manner. However, treating the *Mcu*^−/−^ BMDMs with AZD7545 did not reduce the gene expression of IL-6 and only modestly reduced IL-1β (Extended Data Fig. 7c). These results support the model wherein amplified Ca^2+^ signaling in the cytosol is the main driver of the increased inflammatory output of *Mcu*^−/−^ cells. Although the role of mitochondrial metabolism in regulating this process is not supported by the available data, it cannot be ruled out.

### Abnormal functional polarization of *Mcu*^−/−^ macrophages

Previous reports on the role of mCa^2+^ on macrophage polarization have shown links between mCa^2+^ and macrophage polarization. *Mcu* inhibition is linked to an impaired M2 polarization^44,62^, while deletion of *Mcub* is linked to enhanced M1 polarization and impaired M2 polarization^43^. The M1–M2 polarization is not universally accepted as being physiologically meaningful because macrophages achieve a spectrum of functional states in vivo. Nevertheless, we performed M1 and M2 polarization studies in *Mcu*^−/−^ macrophages (Extended Data Fig. 7d,e). M1 polarized *Mcu*^−/−^ macrophages showed enhanced expression of NF-κB target gene with unexpected expression of Arg1. Interestingly, M2 polarized *Mcu*^−/−^ macrophages also showed enhanced expression of M2 markers. However, such polarization experiments are performed over 24 h and the outputs are an integrated result of many secondary and tertiary signaling cascades in macrophages.

### *Mcu*^−/−^ bone marrow-derived macrophages also show increased NLRP3 inflammasome activation

**Fig. 6:**
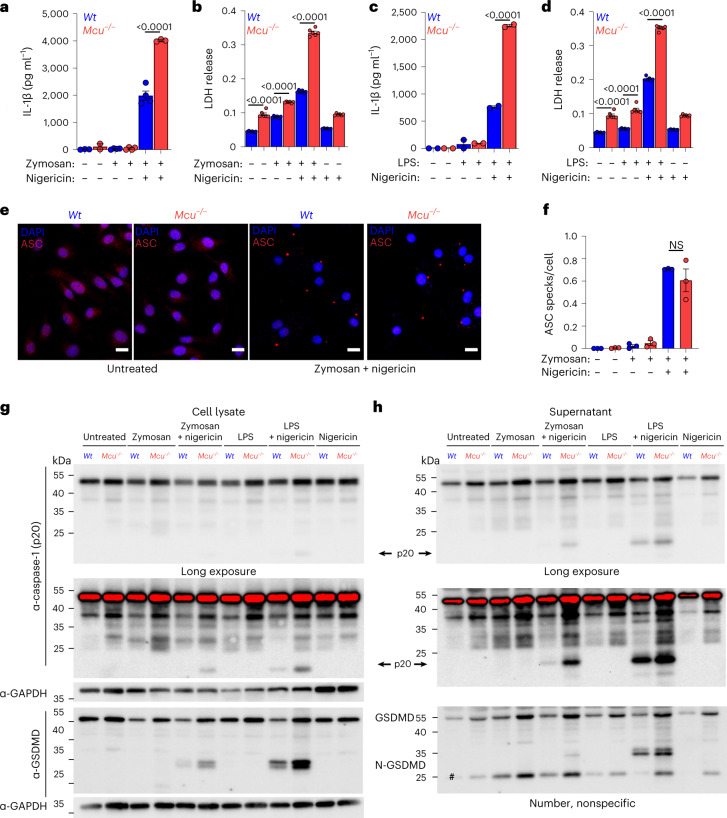
*Mcu*^−/−^ macrophages display increased inflammasome activation and output. **a**, IL-1β enzyme-linked immunosorbent assay (ELISA) of cell supernatants collected from WT and *Mcu*^−/−^ macrophages. Cells were stimulated with zymosan for 3 h followed by nigericin (5 µM) overnight. *N* = 3 biological replicates. Error bars represent the s.e.m.; *P* values were determined by one-way ANOVA. **b**, LDH levels in cell supernatants were collected from WT and *Mcu*^−/−^ macrophages. *N* = 6 biological replicates. Error bars represent the s.e.m.; *P* < 0.0001 determined by one-way ANOVA. **c**, IL-1β ELISA of cell supernatants collected from WT and *Mcu*^−/−^ macrophages. Cells were stimulated with LPS for 3 h followed by nigericin (5 µM) overnight. *N* = 2 biological replicates. Error bars represent the s.e.m.; *P* values were determined by one-way ANOVA. **d**, LDH levels in cell supernatants collected from WT and *Mcu*^−/−^ macrophages. *N* = 6 biological replicates. Error bars represent the s.e.m.; *P* < 0.0001 determined by one-way ANOVA. **e**, Representative images of *Mcu*^−/−^ and WT macrophages immunostained for ASC and nuclei (DAPI). Cells were stimulated with zymosan for 3 h followed by nigericin (5 µM) for 1 h. **f**, Quantification of ASC specks, average number of specks counted per cell, for WT and *Mcu*^−/−^ macrophages. *N* = 3 biological replicates. Error bars represent the s.e.m.; no significance was determined by the one-way ANOVA. **g**, Western blot analysis of cell lysates from WT and *Mcu*^−/−^ macrophages stimulated with zymosan for 3 h followed by nigericin (5 µM) for 1 h. Cell lysates were immunoblotted for caspase-1 (p20), GSDMD and GAPDH. **h**, Western blot analysis of supernatants corresponding to samples shown in **g**. *N* = 1 representative replicate, from two independent experiments. Cell supernatants were immunoblotted for caspase-1 (p20), GSDMD and GAPDH. Source data

**Extended Data Fig. 8:**
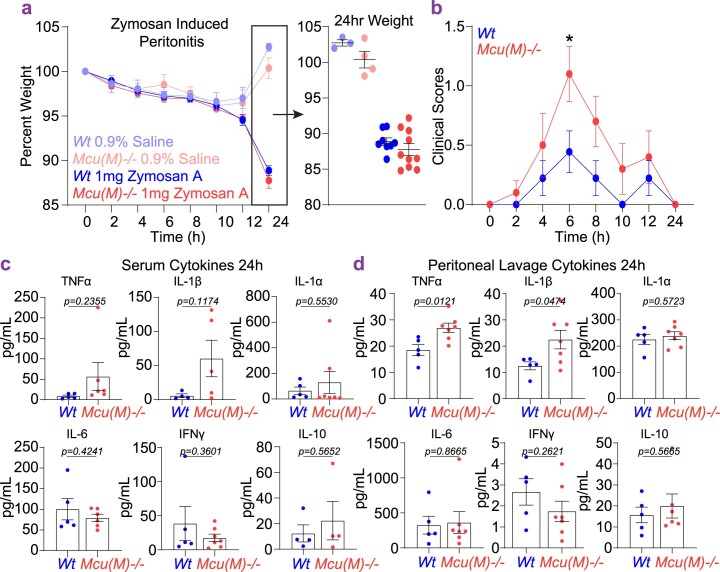
Zymosan induced peritonitis in mice wherein Mcu is deleted selectively in myeloid cells (*Mcu(M)−/−* mice). **a**. Percent weight change for *wt* and *Mcu(M)−/−* mice in a model of Zymosan Induced Peritonitis. N = 9–10 mice. **b**. Clinical Scores for *wt* and *Mcu(M)−/−* mice in a model of Zymosan Induced Peritonitis. N = 9–10 mice. Error bars represent SEM; p≤0.05(*) determined by the one-way ANOVA. **c**. ELISA determined serum cytokines in *wt* and *Mcu(M)−/−* mice, at 24 hours, in a model of Zymosan Induced Peritonitis. N = 5 mice. Error bars represent SEM; *p<0.0001* according to Welch’s t-test, two-tailed. **d**. Peritoneal lavage cytokines at 24 hours. N = 4–5 mice. Error bars represent SEM of two independent experiments: *p-values* according to Welch’s t-test, two-tailed. Source data

Next, we evaluated if the increased inflammatory gene expression caused by decreased mCa^2+^ uptake also increases inflammasome activation. WT and *Mcu*^−/−^ macrophages from young mice were stimulated with zymosan for 3 h before the addition of nigericin (5 µM) for 1 h to activate the NLRP3 inflammasome. *Mcu*^−/−^ macrophages released significantly more IL-1β (Fig. 6a) and lactate dehydrogenase (LDH; Fig. 6b), and this effect was also seen when the macrophages were first stimulated with lipopolysaccharide (LPS; Fig. 6c,d). We wondered if the assembly of the NLRP3 inflammasome is also accentuated in *Mcu*^−/−^ macrophages. Assembly of NLRP3 inflammasome can be visualized through immunofluorescence microscopy of ASC speck formation^63–67^. We did not see any significant difference in ASC speck formation in *Mcu*^−/−^ BMDMs (Fig. 6e,f) indicating that decreased mCa^2+^ uptake, and concomitantly increased cCa^2+^ signaling, does not have a major impact on the assembly of NLRP3 inflammasome. Activation of the NLRP3 inflammasome results in the proteolytic cleavage and activation of caspase-1 (CASP1). Activated CASP1 catalyzes the proteolytic processing of pro-IL-1β to its secreted form IL-1β. CASP1 also cleaves monomeric gasdermin D (GSDMD), thus catalyzing their oligomerization into a large multimeric gasdermin pore in the plasma membrane. The release of many potent pro-inflammatory mediators, including IL-1β and IL-18, is mediated through this large GSDMD pore. Overall, this process results in a highly inflammatory form of cell death called pyroptosis. We assessed the cleavage of CASP1 and GSDMD in NLRP3 activated macrophages. Notably, we found that the cleavage of both CASP1 and GSDMD was significantly increased in *Mcu*^−/−^ macrophages—in both cell pellets (Fig. 6g) and in supernatants (Fig. 6h). These findings indicate that while decreased mCa^2+^ uptake does not affect NLRP3 assembly, it does have a significant impact on the downstream processing of CASP1 and GSDMD. Finally, we evaluated whether deletion of *Mcu* in the myeloid cells would manifest a hyper-inflammatory response in vivo. Previous reports have shown that long exposures to fungal beta-glucans can activate the NLRP3 inflammasome in macrophages^68^. In a model of zymosan-induced peritonitis, mice lacking *Mcu* in the myeloid cells, the *Mcu(M)*^−/−^ mice, exhibited significantly worse clinical scores, and increased levels of IL-1β and tumor necrosis factor (TNF) in the peritoneal cavity (Extended Data Fig. 8a–d). However, we did not see increased levels of other pro-inflammatory cytokines (IL-1α, IL-6 and IFN-γ) that we measured in this model.

### Diminished mitochondrial Ca^2+^ uptake increases inflammatory output

**Fig. 7:**
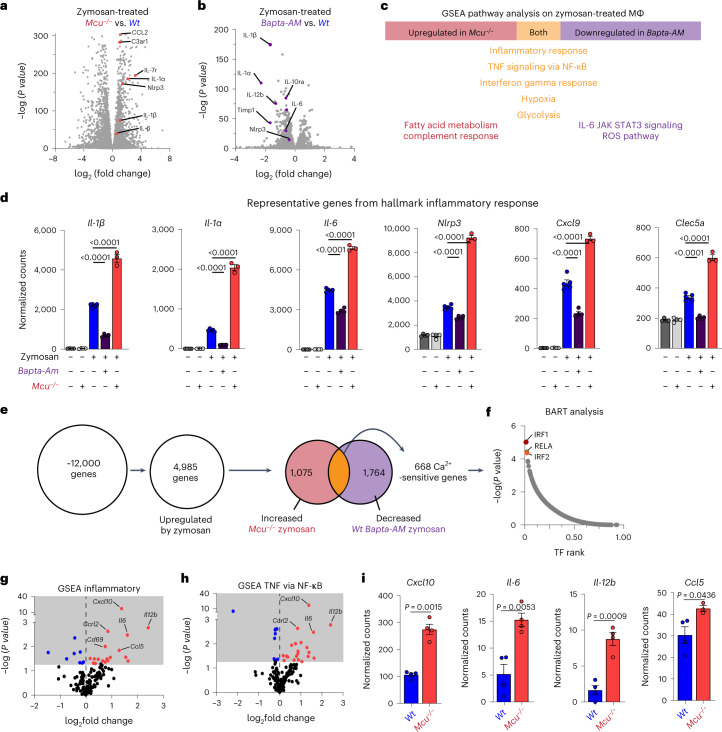
RNA-seq analysis of *Mcu*^−/−^ and BAPTA-AM-loaded BMDMs reveals transcripts sensitive to mCa^2+^ uptake. **a**, Volcano plot showing increased expression of inflammatory genes in *Mcu*^−/−^ BMDMs, compared to WT BMDMs, treated with zymosan for 3 h. Data normalization, dispersion estimates and model fitting (negative binomial) were carried out with the DESeq function (DESeq2 R package). Wald statistics were used for the significance tests. **b**, Volcano plot showing reduced expression of inflammatory genes in BAPTA-AM loaded BMDMs, when compared to unloaded BMDMs, treated with zymosan for 3 h. Data normalization, dispersion estimates and model fitting (negative binomial) were carried out with DESeq. Wald statistics were used for the significance tests. **c**, GSEA pathway analysis of *Mcu*^−/−^, BAPTA-AM loaded (WT) and unloaded WT BMDMs stimulated with zymosan for 3 h. MΦ, macrophage. **d**, Normalized counts for representative genes in the GSEA hallmark inflammatory response pathway. *N* = 3–5 biological replicates. Error bars represent the s.e.m.; *P* values were determined by one-way ANOVA. **e**, Schematic showing gene transcripts used for BART analysis. **f**, BART analysis of 668 mCa^2+^-sensitive genes identified in **a**. Transcription factor (TF) rank was plotted against the −log(*P* value) for each identified transcription factor. **g**, Volcano plot showing gene expression of the GSEA inflammatory response pathway in unstimulated *Mcu*^*−/−*^ macrophages, compared to their WT counterparts. Data normalization, dispersion estimates and model fitting (negative binomial) were carried out with DESeq. Wald statistics were used for the significance tests. **h**, Volcano plot showing expression of genes of the GSEA TNF–NF-κB pathway in unstimulated *Mcu*^*−/−*^ macrophages, compared to their WT counterparts. Data normalization, dispersion estimates and model fitting (negative binomial) were carried out with DESeq. Wald statistics were used for the significance tests. **i**, Normalized counts for representative genes in the GSEA hallmark inflammatory response pathway. *N* = 4 biological replicates. Error bars represent the s.e.m.; *P* value was determined by Welch’s *t*-test, two-tailed. Source data

**Extended Data Fig. 9:**
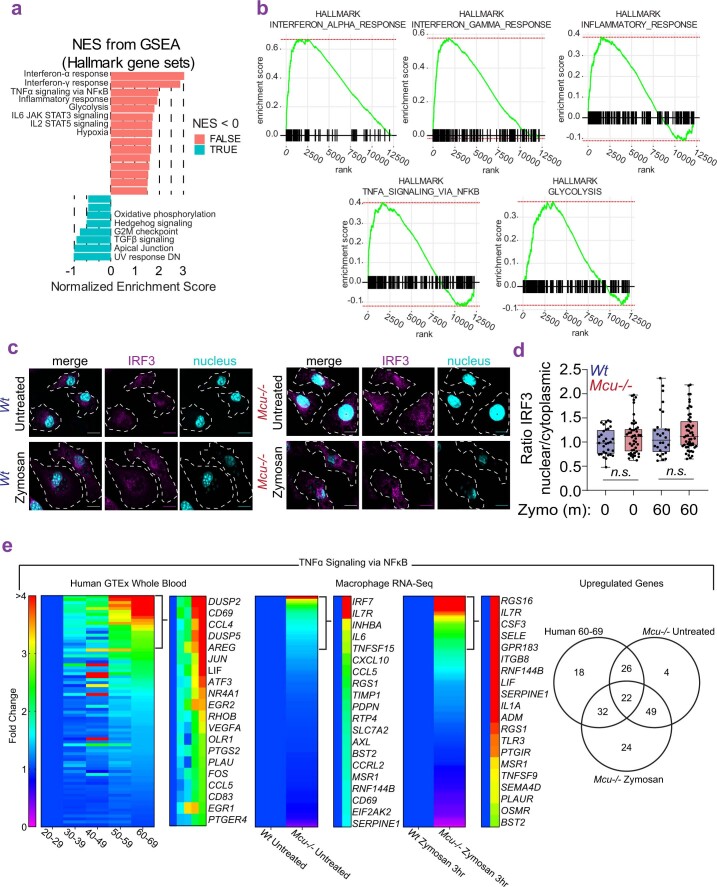
RNA-seq analysis of *Mcu−/−* macrophages and analysis of IRF3 translocation. **a**. GSEA hallmark gene set enrichment analysis based on DSeq2 analysis of *Mcu−/−* versus *wt* BMDMs at baseline (untreated). **b**. Gene set enrichment analysis of GSEA pathways that are differentially expressed in *Mcu−/−* versus *wt* BMDMs at baseline (unstimulated). Enrichment scores (ES) are plotted on y-axis and genes ranked in ordered dataset are plotted in x-axis. **c**. Representative images from *wt* and *Mcu−/−* macrophages, untreated or stimulated with zymosan (30 min) and immunostained for IRF3 and nuclei (DAPI). Outline of cells was determined by bright field image. **d**. Quantified nuclear to cytoplasmic ratios of IRF3 fluorescence intensities in *wt* and *Mcu−/−* macrophages, unstimulated and zymosan-stimulated. N = 35–50 cells, 3 independent experiments. Box and whisker plot represents the min to max values for each data set. Line is at the median; *p value* calculated by one-way ANOVA test (found insignificant). **e**. Heatmaps show comparison of gene expression changes in the TNFα signaling via NFκB pathway from human and mice. Venn-diagram indicates similar genes upregulated in *Human 60–69* compared to young *Mcu−/−* at baseline and following zymosan stimulation. Source data

To develop a systems-level picture of how mCa^2+^ uptake affects the inflammatory response, we performed RNA-seq analysis on WT, *Mcu*^−/−^ and BAPTA-AM loaded macrophages (all derived from young mice), before and after zymosan stimulation. In brief, the experiment was designed to reveal the Ca^2+^-sensitive genes that are dysregulated when mCa^2+^ uptake is diminished. Note that BAPTA-AM loading will affect all Ca^2+^-sensitive genes by ‘clamping’ intracellular Ca^2+^ elevations to near resting levels (<100 nM). We were especially interested in groups of genes that are relatively upregulated in *Mcu*^−/−^ macrophages and downregulated in BAPTA-AM loaded cells. As expected, a volcano plot revealed that many inflammatory genes were significantly upregulated in zymosan-stimulated *Mcu*^−/−^ macrophages when compared to their WT counterparts (Fig. 7a). Conversely, BAPTA-AM loading broadly decreased the expression of inflammatory gene transcription (Fig. 7b). GSEA pathway analysis revealed the key pathways that follow this pattern of regulation in macrophages, that is, upregulated when mCa^2+^ is diminished (cCa^2+^ signaling is enhanced) and downregulated when all Ca^2+^ signaling is prevented by BAPTA-AM (Fig. 7c). Genes involved in inflammatory responses and those involved in the overlapping TNF–NF-κB pathway showed this pattern most clearly. Using normalized counts, we showed significantly increased expression of *Il1b*, *Il1a*, *Il6*, *Nlrp3*, *Cxcl9* and *Clec5a* when mCa^2+^ uptake was diminished during an inflammatory response to zymosan (Fig. 7d). In total, we identified 668 genes that are regulated by mCa^2+^ uptake in zymosan-stimulated macrophages (Fig. 7e). The analysis so far has focused on gene expression changes in response to a potent inflammatory stimulus (zymosan). We checked if abrogation of mCa^2+^ uptake in *Mcu*^−/−^ macrophages upregulates inflammatory genes at baseline—without any overt exposure to an inflammatory stimulus. Surprisingly, although the expression levels were low in quiescent macrophages, we observed a clear upregulation of inflammatory response genes in unstimulated *Mcu*^−/−^ macrophages (Fig. 7g,h). Similar to zymosan stimulation, GSEA pathway analysis revealed a significant enrichment of inflammatory pathways in *Mcu*^−/−^ macrophages at baseline when compared to WT controls (Extended Data Fig. 9a,b). Within the inflammatory pathway genes, *Cxcl10*, *Il6* and *Il12b* were significantly elevated (Fig. 7i). Together, these data show that diminished mCa^2+^ uptake drives low-grade inflammation in the absence of overt inflammatory stimuli and promotes a hyper-inflammatory response when the macrophages are exposed to inflammatory stimuli.

### Analysis of Ca^2+^-sensitive inflammatory gene expression

We applied the binding analysis for regulation of transcription (BART) analysis^69^ to predict transcriptional regulators of the 668 mCa^2+^-sensitive genes we identified (Fig. 7e). This analysis complemented the ex vivo experiments by implicating the RelA family, which includes NF-κB, as being the responsible transcription factors (Fig. 7f). However, this analysis also suggested that transcription by interferon regulatory transcription factor (IRF) family proteins IRF1 and IRF3 is regulated by mCa^2+^ uptake; however, unlike NF-κB, we did not find any enhancement of IRF3 translocation in *Mcu*^−/−^ cells (Extended Data Fig. 9c,d). It is, however, possible that Ca^2+^ signaling regulates an ancillary process of IRF3-mediated gene transcription. When we compare these datasets for similarities across species, we can identify 22 genes associated with inflammaging and reduced mCa^2+^ uptake in macrophages (Extended Data Fig. 9e).

### *Mcu* knockdown in human macrophages increases inflammatory output

**Fig. 8:**
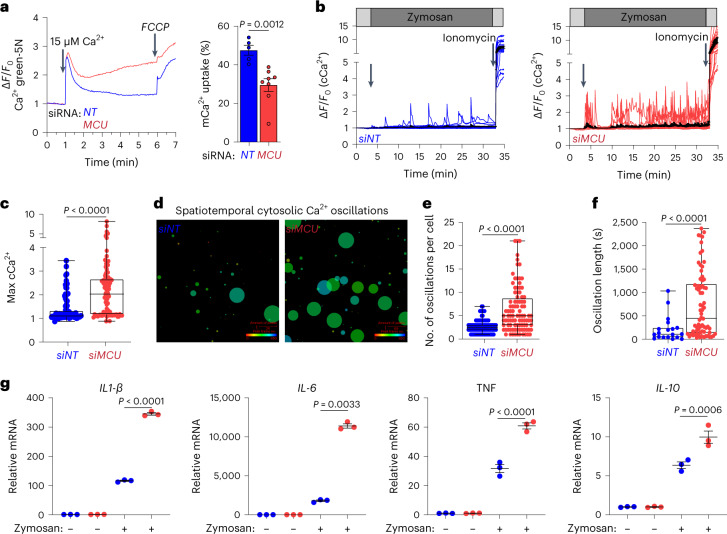
siRNA-mediated depletion of *MCU* in human monocyte-derived macrophages renders them hyper-sensitive to inflammatory stimuli. **a**, Representative trace for a mCa^2+^ uptake in permeabilized *siNT* and *siMCU*-transfected HMDMs. Right, quantification of mCa^2+^ uptake. *N* = 5–8 biological replicates. Error bars represent the s.e.m.; *P* = 0.0012 determined by *t*-test. **b**, cCa^2+^ oscillations in *siNT* and *siMCU*-transfected HMDMs. Δ*F/F*_0_ values for Fura-2-AM loaded macrophages were plotted. Images were taken every 3 s. **c**, Maximum cCa^2+^. *N* = 88–98 cells, three independent experiments. Whiskers represent the minimum to maximum values for each dataset. The box represents the 75th and 25th percentiles. The line is the median; *P* < 0.0001 according to Welch’s *t*-test, two-tailed. **d**, CALIMA maps depicting spatiotemporal aspects of cCa^2+^ elevations. **e**, Oscillation frequency was determined for individual cells. *N* = 88–98 cells, three independent experiments. Whiskers represent the minimum to maximum values for each dataset. The box represents the 75th and 25th percentiles. The line is at the median; *P* < 0.0001 according to Welch’s *t*-test, two-tailed. **f**, Oscillation length was determined for individual cells. *N* = 88–98 cells, three independent experiments. Whiskers represent the minimum to maximum values for each dataset. The box represents the 75th and 25th percentiles. The line is the median; *P* < 0.0001 according to Welch’s *t*-test, two-tailed. **g**, Gene expression of IL-1β, IL-6, TNF and IL-10 mRNA. *N* = 3 biological replicates. Error bars represent the s.e.m.; *P* values were determined by one-way ANOVA. Source data

**Extended Data Fig. 10:**
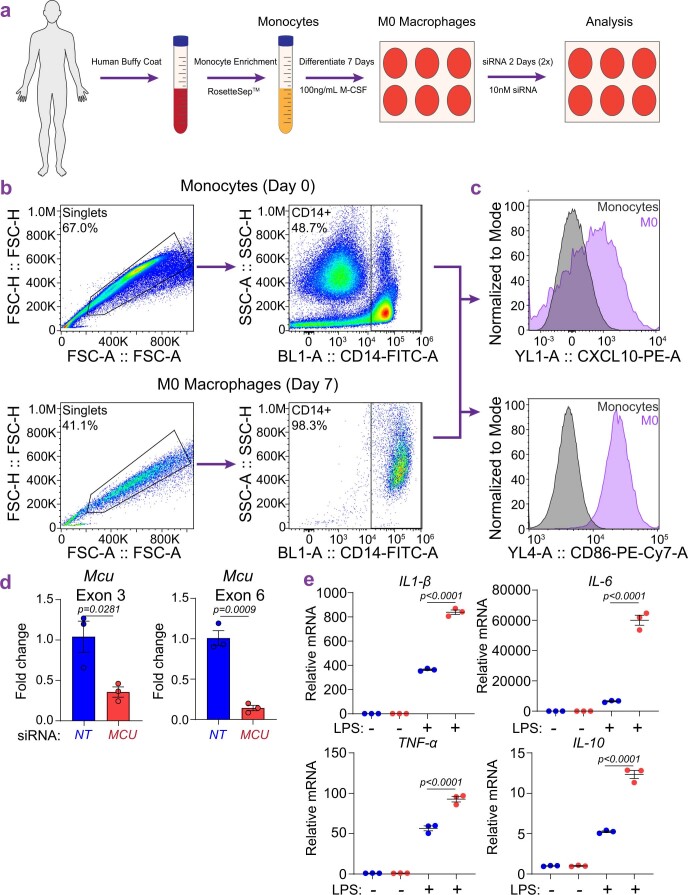
Validation of HMDMs and analysis of LPS response in HMDMs. **a**. Schematic for isolation and differentiation of HMDMs from human buffy coats. **b**. Flow cytometry-based validation of enriched human monocytes and differentiated HMDMs. **c**. Upregulation of CXCL10 and CD86 in HMDMs after differentiation from monocytes. **d**. *MCU* mRNA levels in *siNT* and *siMCU-transfected* HMDMs. N = 3 biological replicates. Error bars represent SEM; *p values* determined by unpaired t-test. **e**. Fold changes in the gene expression of IL-1β, IL-6, TNFα, and IL-10 mRNA. N = 3 biological replicates. Error bars represent SEM; *p-values* determined by the one-way ANOVA. Source data

As illustrated (Extended Data Fig. 10a), we differentiated human monocyte-derived macrophages (HMDMs) by first enriching the human monocytes from donor buffy coats and then culturing them for 7 d in growth medium supplemented with macrophage colony-stimulating factor. Flow cytometry analysis confirmed proper differentiation of the HMDMs. The HMDMs exhibited high-density staining of the macrophage markers CXCL10 and CD86, which were absent on undifferentiated monocytes on day 0 (Extended Data Fig. 10b,c). The short interfering RNA (siRNA)-mediated knockdown of *MCU* successfully reduced the mRNA levels of *MCU*, as measured by quantitative PCR with reverse transcription (RT–qPCR) analysis of *MCU* exon 3 and exon 6 (Extended Data Fig. 10d). Comparison of the mCa^2+^ uptake capacity clearly showed a robust uptake in HMDMs transfected with scrambled siRNA control (*siNT*-HMDMs) and a significantly diminished mCa^2+^ uptake in HMDMs transfected with MCU siRNA (*siMCU*-HMDMs; Fig. 8a). When stimulated with zymosan, the control *siNT*-HMDMs displayed Ca^2+^ oscillations throughout the 30 min of imaging. But similar to BMDMs-O and *Mcu*^−/−^ BMDMs, the *siMCU*-HMDMs showed a significant increase in both the frequency and amplitude of the Ca^2+^ oscillations (Fig. 8b,c). The spatiotemporal analysis of the Ca^2+^ oscillations also revealed a similarity to mouse WT BMDMs-O and *Mcu*^−/−^ BMDMs-Y. The *siMCU*-HMDMs exhibited significantly more Ca^2+^ spikes on an individual basis (Fig. 8d–f). Then, we checked the inflammatory response in *siMCU*-HMDMs and found that the expression levels of pro-inflammatory cytokines IL-1β, IL-6 and TNF were significantly higher when compared to *siNT*-HMDMs (Fig. 8g). Similar results were seen with LPS stimulation (Extended Data Fig. 10e). These results show that the sensitivity of the inflammatory response to mCa^2+^ uptake is conserved and can be demonstrated readily in human macrophages.

## Discussion

In this study, we report a surprising discovery that mCa^2+^ uptake capacity in macrophages drops significantly with age. This amplifies cCa^2+^ signaling and promotes NF-κB activation, rendering the macrophages prone to chronic low-grade inflammatory output at baseline and hyper-inflammatory when stimulated. Although mitochondrial dysfunction has long been a suspected driver of aging, our study pinpoints the MCU complex as a keystone molecular apparatus that links age-related changes in mitochondrial physiology to macrophage-mediated inflammation.

Gene expression analyses of human blood revealed clear signs of chronic age-associated inflammation, supporting the idea that blood transcriptomics can be used to monitor biomarkers of age-related low-grade inflammation. Both chronic low-grade inflammation and mitochondrial dysfunction are known hallmarks of aging, but mechanistic links between these two processes have not been defined with clear links to human biology^70,71^. For example, defective mitophagy in *Prkn*^−/−^ mice may contribute to inflammaging by shedding mitochondrial DNA as an inflammatory stimulus in senescent cells^71^. Although a progressive age-associated decline in mitophagy is not evident in human myeloid cells, if one supposes that there is a steady age-associated shedding of inflammatory mediators from other senescent cells, our findings predict that the decreased mCa^2+^-uptake capacity will render the macrophages hyper-responsive to such inflammatory stimuli from senescent cells and thereby drive inflammaging. A recent study performed a comprehensive analysis of mitochondrial phenotypes in purified human cell types and mixtures but omitted mCa^2+^ uptake as a marker of mitochondrial fitness^72^. Interestingly, the authors found that their mitochondrial health index was most impaired in monocytes isolated from aged human donors. Although we chose to focus on macrophage-mediated inflammation, the broad outlines of the mechanistic model are likely applicable to other myeloid cells such as neutrophils and mast cells too, and that is an important line for our future investigations.

Whether macrophages deficient in mCa^2+^ uptake are hyper-inflammatory to all kinds of inflammatory stimuli is an outstanding question. For example, tissue-resident macrophages are constantly exposed to purinergic signals^73^ and our model predicts that in older mice and humans, the reduced Ca^2+^-buffering capacity of mitochondria will render the myeloid cells hyper-responsive to such purinergic signals. In addition to increased inflammation, a reduction in mCa^2+^ uptake also affects the ability of macrophages to phagocytose and kill pathogens, an immunological deficit known to be related to age. Analysis of genes that are especially sensitive to mCa^2+^ uptake points to the RelA family as the key transcription factors involved in this process. Consistently, we show that the nuclear translocation of NF-κB, which is central to the inflammatory response, is enhanced when mCa^2+^ uptake is diminished. Notably, the loss of mCa^2+^ uptake also results in hyperactivation of NFAT^74^, a transcription factor that is exquisitely sensitive to Ca^2+^ signaling. But in contrast to NFAT regulation, where Ca^2+^ is known to regulate NFAT nuclear translocation through the Ca^2+^-activated phosphatase calcineurin^75,76^, the precise mechanisms through which Ca^2+^ regulates NF-κB translocation are not entirely clear.

In a landmark study, Fieni et al. used direct patch-clamp recordings of mitoplasts to demonstrate that MCU current density varies greatly between tissues^77^. Remarkably, they found that MCU current densities in the cardiac mitoplasts from newborn mice were nearly five times larger than those found in the adult counterparts. In accordance with those findings, our analysis of the human gene expression data shows that expression of *MCU* in the heart reduces progressively with age. In addition to age-related changes in gene expression, other ancillary mechanisms, such as posttranslational modifications of the MCU complex, are likely to be very relevant to the overall regulation of mCa^2+^ uptake. Although we have clearly demonstrated that reduced mCa^2+^ uptake is an important component of this aging process, we cannot rule out other age-associated changes in the molecular machinery of Ca^2+^ signaling. For instance, we have evidence that *ITPR3* expression increases with age. Interestingly, it was proposed recently that loss of *Itpr2* leads to improved lifespan in mice^55^. Our future studies will focus on determining whether the ER–mitochondria contact sites in the macrophages are disrupted with age and on defining the regulatory mechanisms of the age-associated reduction in *MCU* transcription.

A major paradox in the field of mCa^2+^ signaling is that despite that its machinery is highly conserved in invertebrates and vertebrates^78^, and expressed ubiquitously in mammalian tissues, the deletion of *Mcu*, in the mixed background, yields smaller but viable mice^79^. These mice display moderate defects in skeletal muscle function^79^, but the overall phenotype is surprisingly mild for a process that is so well conserved and ubiquitous. The phenotype of global *Mcu*^−/−^ mice is likely confounding in this respect because when a gene is deleted during embryogenesis, there is often a developmental compensation (not necessarily in the same molecular function). It is now becoming increasingly clear that a major role for mCa^2+^ signaling is tied to innate immune responses, the salience of which is largely masked in unchallenging conditions of a mouse vivarium. Recent studies establish that far from being a redundant Ca^2+^-buffering system, this molecular apparatus, centered on MCU and its regulatory subunits, has a profound role in host defense and inflammatory processes. Ironically, a steady age-associated erosion of its activities not only dampens the innate immune responses to fungal pathogens^42^, but also drives chronic low-grade inflammation. Interestingly, *MCU*-mutant flies show reduced lifespan^80^ but analyses of MCU-null hemocytes, the cells that constitute the *Drosophila* innate immune system, were not carried out. In mammals, tissue-resident macrophages occupy specialized niches in all organ systems. Age-associated decrease in the mCa^2+^-uptake capacity in these specialized tissue-resident macrophages may increase local inflammation and thereby have a major impact on organ physiology and homeostasis. An intriguing possibility is that resident macrophages of certain organ systems may be especially susceptible to such age-related changes. Our study sets the stage for many such research directions that may ultimately allow us to slow the onset and progression of many age-related diseases where chronic inflammation plays either a germinating or exacerbating role.

## Methods

### Mouse strains

Male and female mice aged 15 to 25 weeks (young) and 80–90 weeks (old) were used for all experiments. C57BL/6 mice we purchased from Jackson Laboratories (000664) within indicated age ranges. For WT and *Mcu(M)*^*−/−*^ mouse experiments, mice aged 15–25 weeks were used. *Mcu(M)*^*−/−*^ mice are reported previously^42^. The mouse experiments carried out in this study were reviewed and approved by the University of Virginia IACUC under the active protocol 3916.

### Cell lines and cell culture

All cells were grown and maintained at 37 °C, 5% CO_2_. BMDMs were isolated and cultured as previously described^81^. In brief, bone marrow was extracted from mouse femur and tibia via centrifugation. The red blood cells were lysed with ACK lysis buffer and the remaining cells were counted and plated on petri dishes at a density of 2–4 × 10^6^ cells per plate in BMDM Media (RPMI 1640 + 10% FBS + 20% L929-conditioned media). Cells were differentiated for 7 d and medium was replaced every 3 d. For experiments, BMDMs were used between days 9–14 after harvest.

### GTEx and differential gene expression analysis

GTEx Analysis V8, gene counts and metadata were downloaded from the GTEx portal (https://gtexportal.org/)^49^ and analyzed using RStudio. Expression profile data were obtained for different tissues, binned into age groups and then subjected to differential gene expression analysis using DESeq2 (ref. ^82^) R package. PCA plots were generated using the plotPCA function. The differentially expressed genes were ranked based on the log_2_ fold change and FDR-corrected *P* values. The ranked list was then used to perform pathway analysis using GSEA software^83^. For the analysis of genes associated with mitochondrial functions, the differentially expressed genes were uploaded to mitoXplorer1.0 (ref. ^50^) for pathway analysis. Comparative plots were generated for specified pathways and the log_2_ fold change was plotted for individual genes.

### Mitochondrial Ca^2+^ uptake in permeabilized macrophages

Ca^2+^ uptake assay was adapted for macrophages from Wettmarshausen et al.^84^ as reported in Seegren et al.^42^. In brief, cells were washed two times in D-PBS (without Ca^2+^ and Mg^2+^) and resuspended in ICM buffer containing 120 mM KCl, 5 mM NaCl, 1 mM MgCl_2_, 2 mM KH_2_PO_4_, 20 mM HEPES, 5 mM succinate, 5 mM malate, 5 mM glutamate, 500 nM thapsigargin and 0.1 µM calcium green-5N. Cells were immediately permeabilized with 35 µM digitonin for 5 min before recording on a FlexStation plate reader. Calcium green-5N fluorescence intensity was recorded every 2 s for the indicated time with injections of CaCl_2_ (concentration indicated in figure legend) and 10 µM FCCP at indicated times.

### Cytosolic Ca^2+^ imaging using ratiometric Fura-2

Ratiometric imaging of macrophages using Fura-2-AM was as described previously by Seegren et al.^42^. In brief, macrophages were incubated for 30 min with gentle agitation at room temperature (RT) with 5 μM Fura-2-AM, 0.02% of pluronic acid and 500 μM probenecid in Ringer solution (155 mM NaCl, 4.5 mM KCl, 2 mM CaCl_2_, 1 mM MgCl_2_, 5 mM HEPES and 10 mM glucose, pH 7.4). Fura-2 emissions were collected at 510 nm and with 340/380 nm of excitation. Excitation was performed using a DG4 Illuminator (Sutter Instruments).

### CALIMA analysis

Images acquired from cCa^2+^ imaging were uploaded into the CalciumImagingAnalyser from Radstake et al.^53^. Regions of interest were drawn manually over cells and processed for recorded cell activity. Spike detection parameters were set to the same values for each replicate and Excel sheets were exported for analysis in PRISM.

### Bulk RNA-seq analysis

On average we received 30 million paired-end sequences for each of the replicates. RNA-seq libraries were checked for their quality using the fastqc program (http://www.bioinformatics.babraham.ac.uk/projects/fastqc/). The results from fastqc were aggregated using MultiQC software^85^. A program developed in-house was used for adaptor identification, and any contamination of adaptor sequence was removed with cutadapt (https://cutadapt.readthedocs.io/en/stable/). Reads were then mapped with the ‘splice aware’ aligner ‘STAR’^86^, to the transcriptome and genome of mm10 genome build. The HTseq software^87^ was used to count aligned reads that map onto each gene. The count table was imported to R to perform differential gene expression analysis using the DESeq2 package^82^. Lowly expressed genes (genes expressed only in a few replicates and had low counts) were excluded from the analysis before identifying differentially expressed genes. Data normalization, dispersion estimates and model fitting (negative binomial) were carried out with the DESeq function. The differentially expressed genes were ranked based on the log_2_fold change and FDR-corrected *P* values. The ranked file was used to perform pathway analysis using GSEA software^83^. The enriched pathways were selected based on enrichment scores as well as normalized enrichment scores.

### Binding analysis for regulation of transcription

A gene list of 668 Ca^2+^-sensitive genes was uploaded into the BART web interface developed and maintained by the C. Zang laboratory at the University of Virginia^69^. The software identified the most likely transcription factors regulating the input genes. The area under the curve and *P* values were exported and plotted.

### Immunoblotting

#### For analysis of caspase-1 and GSDMD

After treatment, the plates were centrifuged at 400*g* for 4 min and cell-free supernatants were collected. Cell lysates were prepared by directly adding 1× Laemmli sample buffer into the pellets and stored at −80 °C. At the day of electrophoresis, cell lysates were transferred into a 1.5-ml tube, sonicated and boiled at 95 °C for 5 min. Collected supernatants were cleared again by centrifugation at 400*g* for 5 min. Proteins in the supernatants were precipitated using 20% trichloroacetic acid, resuspended in 1× Laemmli sample buffer, and boiled at 95 °C for 5 min. Cell lysates and concentrated supernatants were run on a 12% homemade SDS–PAGE gel and transferred on to a 0.45-µm PVDF membrane (Millipore) using Towbin wet transfer buffer. After transfer, Ponceau S staining was performed to confirm equal loading of total proteins and the membrane was then blocked by 5% non-fat milk in TBST for 1 h at RT. Primary antibodies were diluted in TBST and incubated at 4 °C overnight. Horseradish peroxidase (HRP)-conjugated secondary antibodies were diluted in TBST at 1:10,000 and incubated for 1 h at RT. Membrane was developed by adding Luminata Forte Western HRP substrate (Millipore, WBLUF0100) and imaged on a Bio-Rad ChemiDoc Imager. Primary antibodies used were mouse anti-mCasp1(p20) (AdipoGen, Casper-1, 1:1,000 dilution) and rabbit anti-mGSDMD (Abcam, ab209845; 1:1,000 dilution). Secondary antibodies used were anti-mouse, HRP (Jackson, 115-035-003) and anti-rabbit, HRP (Jackson, 111-035-144).

### Nuclear and cytoplasmic fractions

The Thermo Scientific NE-PER Nuclear Cytoplasmic Extraction Reagent kit was used to generate nuclear and cytosolic fractions from cells following zymosan stimulation. Briefly, cells underwent reagent-based lysis using cytoplasmic extraction reagents I & II followed by centrifugation for 5 min at 16,000*g* to separate nuclei from cytosolic fractions. The nuclei were then lysed using the nuclear extraction reagent and centrifuged for 5 min at 16,000*g*. The resulting supernatant contained the nuclear extract and was used for subsequent western blotting.

### Native gel electrophoresis of MCU complex

The mitochondrial membrane proteins were extracted by incubating the isolated mitochondria with 1% digitonin on ice for 30 min. Samples were vortexed every few minutes. The mitochondrial extracts were then mixed at a 1:1 ratio with non-reducing sample loading buffer (62.5 mM Tris-HCl, pH 6.8, 40% (wt/vol) glycerol and 0.01% (wt/vol) bromophenol blue). Samples were resolved on 4–20% Mini-Protean TGX Stain-Free precast gels (Bio-Rad; 4568096) and run at 200 V for 5 h on ice. The gels were transferred onto a PVDF membrane using the Bio-Rad Turbo-blot system. Membranes were blocked with 5% milk for 30 min with gentle agitation before immunoblotting with primary antibody (α-MCU, clone D2Z3B, Cell Signaling 14997S and α-MICU1, clone D4P8Q, Cell Signaling 12524) in Signal Boost Immunoreaction Enhancer (Calbiochem, 407207-1KIT) at a 1:1,000 dilution. Staining with primary antibody was carried out overnight at 4 °C. Secondary anti-rabbit HRP was used in Signal Boost Immunoreaction enhancer for 2 h. The immunoblots were developed with SuperSignal West Pico PLUS Chemiluminescent Substrate (Thermo Fisher, 34580) 5 min before imaging.

### qPCR

For all qPCR experiments, macrophages were plated at ~80% confluency into tissue culture-treated plates and rested overnight. For inflammatory gene expression, the following day, BMDMs were stimulated with Zymosan A BioParticles (Thermo Fisher, Z2849) at two particles per cell. For pharmacological pretreatments, on the day of experiment, BMDMs were treated with inhibitors for 30 min before the addition of zymosan, as described above. Inhibitor information and concentrations used were: BAPTA-AM (Thermo Fisher, B6769; 10 µM), ionomycin (Cayman Chemicals, 10004974; 1 µM), BTP2 (Sigma, 203890-M; 10 µM), zegocractin (MedChemExpress, HY-101942; 1 µM) and AZD7545 (MedChemExpress, HY-16082; 1 µM). For M1 versus M2 polarization, BMDMs were treated either with IFN-γ (R&D Systems, 485-MI-100/CF; 100 ng ml^−1^) and LPS (Invivogen, tlrl-eblps; 100 ng ml^−1^; to induce polarization toward a M1 phenotype) or with IL-4 (R&D Systems, 404-ML-010; 20 ng ml^−1^; to induce polarization toward a M2 phenotype). Total RNA was isolated from treated cells using RNeasy Plus Mini Kit. Following isolation, RNA concentration was determined using a NanoDrop 2000c spectrophotometer. RNA was converted to cDNA in a two-step reverse transcriptase process using the Promega Reverse Transcription Master Mix. Following cDNA synthesis, a Bio-Rad CFX Connect Real-Time system was used to perform qPCR reactions with SYBR Select Master Mix and 1 to 5 ng cDNA per well in a 96-well plate.

### Immunofluorescence

Cells were plated overnight on coverslips before experiments. BMDMs were stimulated with Zymosan A BioParticles (Thermo Fisher, Z2849) at two particles per cell for indicated times. Following treatments, coverslips were washed 3× in PBS to remove loose/non-adherent cells. Coverslips were fixed in 4% paraformaldehyde and 4% sucrose (30 min, RT). Coverslips were washed 3× in wash buffer (PBS with 0.05% Tween-20), blocked and permeabilized at RT for 1 h in B/P buffer (1% BSA, 0.1% Triton X-100 and 0.05% Tween-20 in PBS), and then incubated with primary antibody diluted in B/P buffer overnight at 4 °C. Coverslips were washed 3× in wash buffer, and incubated at RT with the appropriate secondary antibody in B/P buffer for 2 h, followed by 3× washes in wash buffer. Coverslips were mounted on glass slides (ProLong Gold Antifade; Thermo Fisher, P36930), stored in a desiccation box at 4 °C until imaging. Confocal microscopy was performed on a Zeiss LSM 880. Data were acquired with Zen Black and analyzed using ImageJ. Antibodies used for immunofluorescence were: anti-ASC/TMS1/PYCARD antibody (F-9): sc-271054, Santa Cruz; anti-NF-κB p65 (D14E12) XP rabbit monoclonal antibody 8242, Cell Signaling; anti-IRF-3 (D83B9) rabbit monoclonal antibody 4302, Cell Signaling.

### Mitogenie

To analyze mitochondrial morphology and other characteristics, images were cropped into individual cells and processed using a mitochondrial analysis workflow developed by the Kashatus laboratory^52^. Images were first input into the MitoCatcher application on the Mitogenie platform, generating binarized images of segmented mitochondrial networks. The MiA application on Mitogenie was used to analyze the images of the mitochondrial networks and produce quantitative measurements describing mitochondrial morphology.

### LDH assay

BMDMs were incubated for 3 h with LPS (Invivogen, tlrl-eblps; 100 ng ml^−1^) or Zymosan A BioParticles (Thermo Fisher, Z2849; two particles per cell) at 37 °C and 5% CO_2_. After 3 h, cells were washed 3× with HBSS, resuspended in Ringer solution (155 mM NaCl, 4.5 mM KCl, 2 mM CaCl_2_, 1 mM MgCl_2_, 5 mM HEPES and 10 mM glucose, pH 7.4) with or without nigericin (Invivogen, tlrl-nig; 5 µM). After 24 h, LDH was measured in the supernatants using Pierce LDH Cytotoxicity Assay Kit (Thermo Fisher Scientific) according to the manufacturer’s instructions.

### IL-1β ELISA

BMDMs were incubated for 3 h with LPS (Invivogen, tlrl-eblps; 100 ng ml^−1^) or Zymosan A BioParticles (Thermo Fisher, Z2849; two particles per cell) at 37 °C, 5% CO_2_. After 3 h, cells were washed 3× with HBSS, resuspended in Ringer solution (155 mM NaCl, 4.5 mM KCl, 2 mM CaCl_2_, 1 mM MgCl_2_, 5 mM HEPES, 10 mM glucose, pH 7.4) with or without nigericin (Invivogen, tlrl-nig; 5 µM). After 24 h, IL-1β was measured in the supernatants using ELISA MAX Standard Mouse IL-1β (BioLegend) according to the manufacturer’s instructions.

### Zymosan-induced peritonitis

*Mcu(M)*^−/−^ and WT mice were subjected to a model of zymosan-induced peritonitis^88^. In brief, mice were intraperitoneally injected with 55 mg per kg body weight Zymosan A and monitored for 24 h for clinical scores of conjunctivitis, lethargy, changes in hair coat and grimace pain to indicate symptoms of illness. Weight was monitored every 2 h and scoring was performed by a blinded member of the laboratory. Following 24 h, mice were euthanized. Blood and peritoneal lavage fluid were collected for Luminex analysis and cytokine detection.

### Differentiation of human monocyte-derived macrophages

Human monocytes were isolated from healthy donor buffy coats procured from the American Red Cross Biomedical Services (ARCBS), with consent from the volunteer donors for whole-blood collection and its use for research (Leukpacks/Whole Blood Clinical Study Protocol LP-2). The distribution of buffy coats to the laboratory of B.N.D. at University of Virginia was also reviewed and approved by the ARCBS Institutional Review Board under the active protocol 2016-030. Differentiation of HMDMs was performed using PromoCell, Serum-free and Zeno-free cell culture method. In brief, buffy coats were enriched for monocytes using RosetteSep Human Monocyte Enrichment Cocktail. Enriched monocytes were plated on six-well plates in monocyte attachment medium for 1 h in a 5% CO_2_ and 37 °C incubator. Cells were washed three times with vigorous swirling in warm monocyte attachment medium to remove non-adherent cells. Cells were cultured for 7 d in Macrophage Generation Medium DXF with supplement mix to generate HMDMs. The siRNA knockdown of *MCU* was performed two times over 48 h using Lipofectamine 3000 with 10 nM siRNA. Antibodies used for flow cytometry were: FITC anti-human CD14 antibody, BioLegend 325603; PE/Cyanine7 anti-human CD86 Antibody, BioLegend 305421; and PE anti-human CXCL10 (IP-10) antibody, BioLegend 519503. Flow cytometry was performed on an Attune NxT with Attune NxT Software (v2.0+).

### Statistics and reproducibility

All data were analyzed using Excel (Microsoft) and Prism 8 (GraphPad) software. All datasets were subjected to ROUT outlier test and the data points with *Q* < 1% were considered outliers and removed. In bar graphs, data are presented as means with error bars reflecting the s.e.m. or as indicated in figure legends. Statistical significance (*P* < 0.05) was computed using one-way ANOVA, two-way ANOVA and Welch’s *t*-test (two-tailed), as indicated in figure legends. The sample size and representation of ‘*n*’ (mice, experimental repeats or cells) is indicated in figure legends. Sample size was determined by using GPower3.l software. For all other experiments (ex vivo and in vivo), no power analysis was used for sample sizes and replicates, but they were determined based on experimental experience. In the box plots, the whiskers represent minimum and maximum values, the box represents the 75th and 25th percentiles and the horizontal line is the median. For zymosan-induced peritonitis, clinical scores were collected by laboratory personnel who were blinded to the genotype/condition of individual mice. All other experiments were not blinded but contained appropriate biological replication. The order of data collection was changed between experiments to avoid collection bias (for example, if the experiment was run as control, WT and then knockout for one experiment, the following experiment data was collected as knockout, WT and then control).

### Reporting summary

Further information on research design is available in the Nature Portfolio Reporting Summary linked to this article.

## Supplementary information

Supplementary Information Supplementary Fig. 1 Unprocessed blots and primer information.

Reporting Summary

## Data Availability

The RNA-seq data are deposited in the publicly available Gene Expression Omnibus database under accession GSE228873. Source data are available with this paper. All other data are available from the corresponding author upon reasonable request.

## Source data

Source Data Fig. 1
Source Data Fig. 2
Source Data Fig. 3
Source Data Fig. 4
Source Data Fig. 5
Source Data Fig. 6
Source Data Fig. 7
Source Data Fig. 8
Source Data Extended Data Fig. 1
Source Data Extended Data Fig. 2
Source Data Extended Data Fig. 3
Source Data Extended Data Fig. 4
Source Data Extended Data Fig. 5
Source Data Extended Data Fig. 6
Source Data Extended Data Fig. 7
Source Data Extended Data Fig. 8
Source Data Extended Data Fig. 9
Source Data Extended Data Fig. 10
