# Supplementary material for: Reduced mitochondrial calcium uptake in macrophages is a major driver of inflammaging
Source: Nat Aging. 2023 Jun 5;3(7):796–812. doi: 10.1038/s43587-023-00436-8 (PMC10353943; doi:10.1038/s43587-023-00436-8)
Supplement: Supplementary file 1 — Unprocessed blots and primer information. [file 43587_2023_436_MOESM1_ESM.pdf]

nature aging

Article https://doi.org/10.1038/s43587-023-00436-8

Reduced mitochondrial calcium uptake in macrophages is a major driver of inflammaging

In the format provided by the authors and unedited

Supplementary Figure 1

Uncropped MICU1 gel for Extended Data Fig. 3a

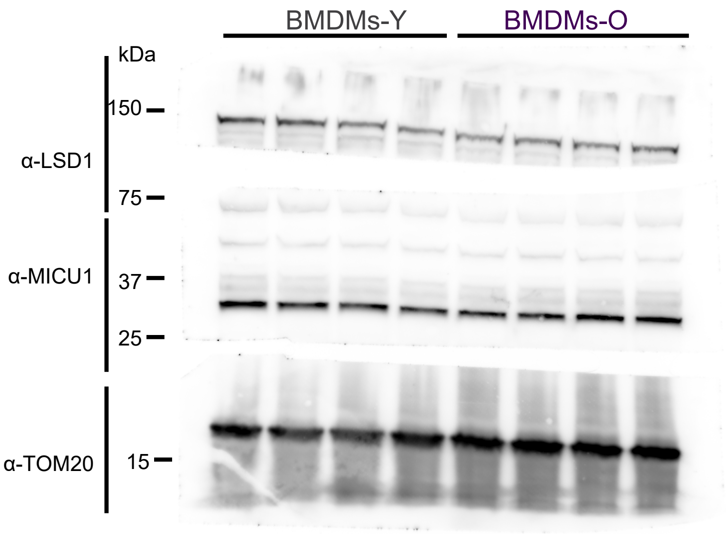

Uncropped MCU Native Gel
Extended Data Fig 3c

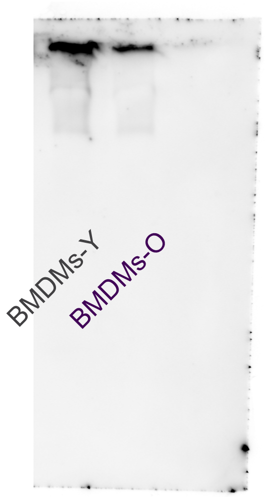

Uncropped Caspase1 gel for Fig. 6g-h

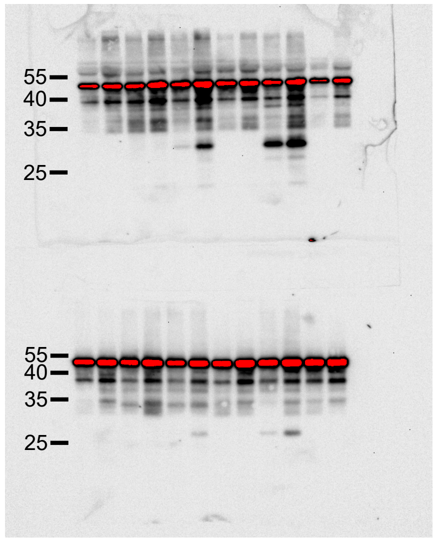

Uncropped GAPDH gel for Fig. 6g-h

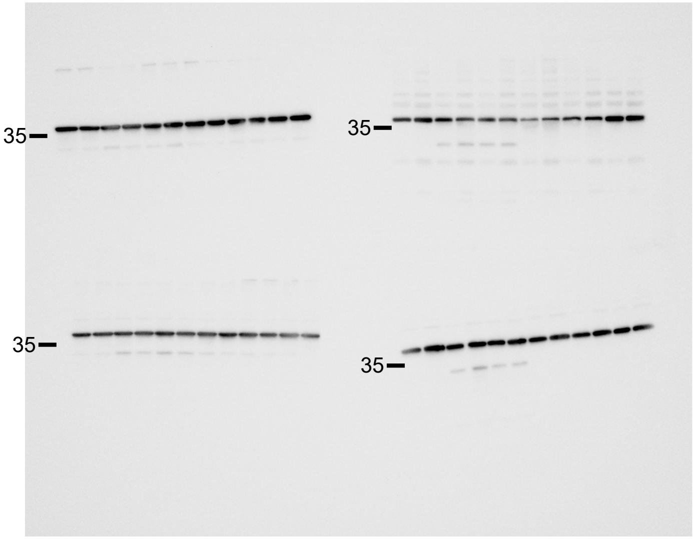

Uncropped GSDMD gel for Fig. 6g-h

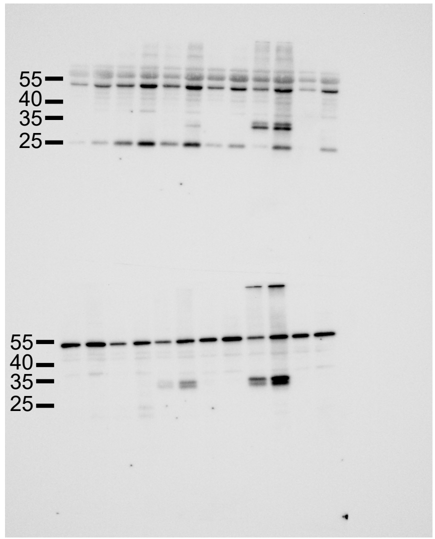

Primers for qPCR

Arg1 Ms Forward (5' – CTGACAACCAGCTCTGGGAA – 3'), Arg1 Ms Reverse (5' – TCCTGGTACATCTGGGAACTTT – 3'), Fizz1 Ms Forward (5' – ACTTCTTGCCAATCCAGCTAAC – 3'), Fizz1 Ms Reverse (5' – CACCCAGTAGCAGTCATCCC – 3'), CD206 Ms Forward (5' – TTCAGCTATTGGACGCGAGG – 3'), CD206 Ms Reverse (5' – GAATCTGACACCCAGCGGAA – 3'), McuB Ms Forward (5' – GCCTTCCCTTGGTAACCCTC – 3'), McuB Ms Reverse (5' – ATTTCACTGCCATCTGCCGT – 3'), EMRE Ms Forward (5' – AGAACTTCGCTGCTCTGCTT – 3'), EMRE Ms Reverse (5' – GCTCCCTGTGCCCTGTTAAT – 3'), Micu2 Ms Forward (5' – AAGGTAGCGACTGGACAGGA – 3'), Micu2 Ms Reverse (5' – TGTGACACCCATAAGCCTCG – 3'), Micu1 Ms Forward (5' – AATTGCCCAGGAACGAGAAA – 3'), Micu1 Ms Reverse (5' – GAGGACTGTTGTGAGGAAGATG – 3'), Mcu Ms Forward 1 (5' – TGAACGACGTGAAGACCCTG – 3'), Mcu Ms Reverse 1 (5' – TTCGTACCTTCTCCAGGGGG – 3'), Mcu Ms Forward 2 (5' – CCCCCTGGAGAAGGTACGAA – 3'), IL-1β Ms Forward (5' - GCAACTGTTCCTGAACTCAACT – 3'), IL-1β Ms Reverse (5' – ATCTTTTGGGGTCCGTCAACT – 3'), iNOS Ms Forward (5' – GTTCTCAGCCAACAATACAAGA – 3'), iNOS Ms Reverse (5' – GTGGACGGGTCGATGTCAC – 3'), IL-6 Ms Forward (5' – TAGTCCTTCCTACCCAATTTCC – 3'), IL-6 Ms Reverse (5' – TTGGTCCTTAGCCACTCCTTC – 3'), TNFα Ms Forward (5' – CCCTCACACTCAGATCATCTTCT – 3'), TNFα Ms Reverse (5' – GCTACGACGTGGGCTACAG – 3'), Itpr1 Ms Forward (5' – CAAGCAACTGCTGGAGGAGA – 3'), Itpr1 Ms Reverse (5' – TTCAAGCTCCTGCTCTGTGG – 3'), Itpr2 Ms Forward (5' – ACAGCAACGTTATCCAACTGC – 3'), Itpr2 Ms Reverse (5' - CAGCATTCACATTATCACCCTCG – 3'), Itpr3 Ms Forward (5' – GGGCCGAAGCCATGAATGAA – 3'), Itpr3 Ms Reverse (5' – CACACTTTGAAGAGGCAATCTCG – 3'), IL-1β Human Forward (5' - TCGCCAGTGAAATGATGGCT – 3'), IL-1β Human Reverse (5' – TGGAAGGAGCACTTCATCTGTT – 3'), IL-6 Human Forward (5' – TTCTCCACAAGCGCCTTC – 3'), IL-6 Human Reverse (5' – GGCGGCTACATCTTTGGAAT – 3'), TNFα Human Forward (5' – CCTGCTGCACTTTGGAGTGA – 3'), TNFα Human Reverse (5' – CAGCTTGAGGGTTTGCTACAAC – 3'), IL-10 Human Forward (5' – TACGGCGCTGTCATCGATTT – 3'), IL-10 Human Reverse (5' – TAGAGTCGCCACCCTGATGT – 3').

siRNA

siRNA used for Mcu knockdown: Dharmacon Cat# L-015519-0-0005, ON-TARGETplus Human MCU (90550) siRNA – SMARTpool, 5nmol. ON-TARGETplus SMARTpool siRNA J-015519-17, MCU Target Sequence: GAUCAGGCAUUGUGGAAUA; ON-TARGETplus SMARTpool siRNA J-015519-18, MCU Target Sequence: GUUUUGACCUAGAGAAAUA; ON-TARGETplus SMARTpool siRNA J-015519-19, MCU Target Sequence: ACUGAGAGACCCAUUACAA; ON-TARGETplus SMARTpool siRNA J-015519-20, MCU Target Sequence: GUAAUGACACGCCAGGAAU.
