# Supplementary material for: Reduced mitochondrial calcium uptake in macrophages is a major driver of inflammaging
Source: Nat Aging. 2023 Jun 5;3(7):796–812. doi: 10.1038/s43587-023-00436-8 (PMC10353943; doi:10.1038/s43587-023-00436-8)
Supplement: Supplementary file 2 — Reporting Summary [file 43587_2023_436_MOESM2_ESM.pdf]

nature portfolio

Corresponding author(s): Bimal Desai

Last updated by author(s): Mar 31, 2023

Reporting Summary

Nature Portfolio wishes to improve the reproducibility of the work that we publish. This form provides structure for consistency and transparency in reporting. For further information on Nature Portfolio policies, see our Editorial Policies and the Editorial Policy Checklist.

Statistics

For all statistical analyses, confirm that the following items are present in the figure legend, table legend, main text, or Methods section.

n/a	Confirmed	
☐	☒	The exact sample size (n) for each experimental group/condition, given as a discrete number and unit of measurement
☐	☒	A statement on whether measurements were taken from distinct samples or whether the same sample was measured repeatedly
☐	☒	The statistical test(s) used AND whether they are one- or two-sided<br>*Only common tests should be described solely by name; describe more complex techniques in the Methods section.*
☒	☐	A description of all covariates tested
☐	☒	A description of any assumptions or corrections, such as tests of normality and adjustment for multiple comparisons
☐	☒	A full description of the statistical parameters including central tendency (e.g. means) or other basic estimates (e.g. regression coefficient) AND variation (e.g. standard deviation) or associated estimates of uncertainty (e.g. confidence intervals)
☐	☒	For null hypothesis testing, the test statistic (e.g. F, t, r) with confidence intervals, effect sizes, degrees of freedom and P value noted<br>*Give P values as exact values whenever suitable.*
☒	☐	For Bayesian analysis, information on the choice of priors and Markov chain Monte Carlo settings
☒	☐	For hierarchical and complex designs, identification of the appropriate level for tests and full reporting of outcomes
☒	☐	Estimates of effect sizes (e.g. Cohen's d, Pearson's r), indicating how they were calculated

Our web collection on statistics for biologists contains articles on many of the points above.

Software and code

Policy information about availability of computer code

Data collection

GTEx Analysis - GTEx Analysis V8 was downloaded from (https://www.gtexportal.org/home/datasets).
Flow cytometry -Attune NxT Software (v2.0 +)
Ca2+ Imaging - Zeiss Axio Observer with DG4 Illuminator and ORCA-Flash 4.0 V2 CMOS camera
IF - LSM880 confocal and a Chameleon multi photon light source
mCa2+ uptake assay - FlexStation 3

Data analysis

GTEx - Expression profile data were obtained for different tissues, binned into age groups and then subjected to differential gene expression analysis using DESeq2 R package. PCA plots were generated using plotPCA function. The differentially expressed genes were ranked based on log2fold change and FDR corrected p-values. The ranked list was then used to perform pathway analysis using GSEA software. For the analysis of genes associated with mitochondrial functions, the differentially expressed genes were uploaded to MitoXplorerl.0 for pathway analysis. Comparative plots were generated for specified pathways and Log2fold change was plotted for individual genes.
Bulk RNAseq - On average we received 30 million paired end for each of the replicates. RNAseq libraries were checked for their quality using the fastqc program (http://www.bioinformatics.babraham.ac.uk/projects/fastqc/). The results from fastqc were aggregated using multiqc software. In house developed programs was used for adaptor identification, and any contamination of adaptor sequence was removed with cutadapt (https://cutadapt.readthedocs.io/en/stable/). Reads were then be mapped with the "splice aware" aligner 'STAR', to the transcriptome and genome of mml0 genome build. The HTseq software will be used to count aligned reads that map onto each gene. The count table was imported to R to perform differential gene expression analysis using the DESeq2 package. Low expressed genes (genes expressed only in a few replicates and had low counts) was excluded from the analysis before identifying differentially expressed genes. Data normalization, dispersion estimates, and model fitting (negative binomial) was carried out with the DESeq function. The log-transformed, normalized gene expression of 50 0 most variable genes will be used to perform an unsupervised principal component analysis. The differentially expressed genes was ranked based on the log2fold change and FDR corrected p-values. The ranked file was used to perform

pathway analysis using GSEA software. The enriched pathways were selected based on enrichment scores as well as normalized enrichment scores.

Image Analysis - All Images were analyzed in ImageJ for nuclear translocation analysis. For Mitogenie: To analyze mitochondrial morphology and other characteristics, images were cropped into individual cells and processed using a mitochondrial analysis workflow developed by the Kashatus lab. Images were first input into the MitoCatcher application on the Mitogenie platform, generating binarized images of segmented mitochondrial networks. The MiA application on Mitogenie was used to analyze the images of the mitochondrial networks and produce quantitative measurements describing mitochondrial morphology.
Data Presentation - All data were analyzed using RStudio, Excel (Microsoft) and Graph Pad Prism 8 (Graph Pad) software. Data are presented as means with error bars which reflect standard error of the mean (SEM) as indicated in figure legends or Box and Whiskers with min, max, 75th, 25th, and median represented as indicated in the figure legend. Statistical significance ($p < 0.05$) was computed using one-way ANOVA, 2-way ANOVA, and welch's t test (two-tailed) as indicated in figure legends. The sample size and representation of 'n' (mice, experimental repeats, or cells) is indicated in figure legends.

For manuscripts utilizing custom algorithms or software that are central to the research but not yet described in published literature, software must be made available to editors and reviewers. We strongly encourage code deposition in a community repository (e.g. GitHub). See the Nature Portfolio guidelines for submitting code & software for further information.

Data

Policy information about availability of data

All manuscripts must include a data availability statement. This statement should provide the following information, where applicable:

- Accession codes, unique identifiers, or web links for publicly available datasets
- A description of any restrictions on data availability
- For clinical datasets or third party data, please ensure that the statement adheres to our policy

Source Data will made available upon request from the Lead Contact, Bimal Desai.
RNAseq data will be made available on gene expression omnibus prior to publication.
Requests for additional information about data should be directed to Lead Contact, Bimal Desai.

Human research participants

Policy information about studies involving human research participants and Sex and Gender in Research.

Reporting on sex and gender	N/A
Population characteristics	N/A
Recruitment	N/A
Ethics oversight	N/A

Note that full information on the approval of the study protocol must also be provided in the manuscript.

Field-specific reporting

Please select the one below that is the best fit for your research. If you are not sure, read the appropriate sections before making your selection.

☒ Life sciences ☐ Behavioural & social sciences ☐ Ecological, evolutionary & environmental sciences

For a reference copy of the document with all sections, see nature.com/documents/nr-reporting-summary-flat.pdf

Life sciences study design

All studies must disclose on these points even when the disclosure is negative.

Sample size	For Zymosan-Induced Peritonitis sample size was determined using GPower3.l software.<br>For all other experiments (Ex vivo and In vivo) sample sizes are indicated in the figure legends and/or listed within the figure panel. Statistical analyses are described in the figure legend. No power analysis was used for sample sizes and replicates, but were determined based on experimental experience.
Data exclusions	The ROUT Outlier Test was performed on datasets. Outliers were determine with Q = 1% for all ROUT tests performed. Outliers were removed from datasets prior to graphing.

Replication	Replication is indicated in figure legend or figure where applicable. In general, all experiments were run in with biological replicates. When representative data is shown, individual data points and/or means of the independent samples are shown, as described in the figure legends.
Randomization	Aged mice were randomly placed into groups when applicable. In ex vivo experiments control and test wells were randomly assigned for each experimental repeat.
Blinding	For Zymosan-Induced Peritonitis clinical scores were collected by lab personal who were blinded to the genotype/ condition of individual mice. All other experiments were not blinded but performed in appropriate biological replication by independent personal to avoid bias.

Reporting for specific materials, systems and methods

We require information from authors about some types of materials, experimental systems and methods used in many studies. Here, indicate whether each material, system or method listed is relevant to your study. If you are not sure if a list item applies to your research, read the appropriate section before selecting a response.

Materials & experimental systems

n/a	Involved in the study
☐	☒ Antibodies
☒	☐ Eukaryotic cell lines
☒	☐ Palaeontology and archaeology
☐	☒ Animals and other organisms
☒	☐ Clinical data
☒	☐ Dual use research of concern

Methods

n/a	Involved in the study
☒	☐ ChIP-seq
☐	☒ Flow cytometry
☒	☐ MRI-based neuroimaging

Antibodies

Antibodies used	TOM20 Polyclonal Antibody (Thermo Scientific, 11802-1-AP), NF-kB p65 (clone:D14E12, CST #8242), IRF-3 (clone:D83B9, CST, #4302), MCU (clone:D2Z3B, CST#14997), MICU1 (clone:D4P8Q, CST#12524), FITC anti-human CD14 (Biolegend, 325603). PE anti-human CXCL10 (IP-10) (Biolegend, 519503), PE/Cyanine7 anti-human CD86 (Biolegend, 205421), TruStain FcX anti-mouse CD16/32 (Biolegend 101320)
Validation	No validation reported from manufacturers.

Animals and other research organisms

Policy information about studies involving animals; ARRIVE guidelines recommended for reporting animal research, and Sex and Gender in Research

Laboratory animals	Old and Young Mice - Male and female mice aged 15-25 weeks (young) and 80-90 weeks (old) were used for all age-related experiments.<br>C57BL/6 mice were purchased from Jackson Laboratories (Stock: 000664) within indicated age ranges.<br>Mcu(M)-/- and WT - Mcu(M)fl/fl Cx3cr1 cre mice were generated by crossing B6;129S-Mcutm1.1jmol/J (Jackson Laboratories; 029817) mice to B6J.B6N(Cg)-Cx3cr1tm1.1(cre)Jung/J (Jackson Laboratories; 025524).
Wild animals	No wild animals used
Reporting on sex	No sex specific differences reported or observed
Field-collected samples	No field-collected samples
Ethics oversight	Mice were housed and bred in accordance with policies and procedures of the University of Virginia Animal Care and Use Committee (IACUC)

Note that full information on the approval of the study protocol must also be provided in the manuscript.

Flow Cytometry

Plots

Confirm that:

☒ The axis labels state the marker and fluorochrome used (e.g. CD4-FITC).

☒ The axis scales are clearly visible. Include numbers along axes only for bottom left plot of group (a 'group' is an analysis of identical markers).

☒ All plots are contour plots with outliers or pseudocolor plots.

☒ A numerical value for number of cells or percentage (with statistics) is provided.

Methodology

Sample preparation	Human buffy coats were collected by the American Red Cross for isolation of human monocytes and differentiation of macrophages. Differentiation of HMDMs was performed using PromoCell, Serum-free and Zeno-free cell culture method.
Instrument	Life Technologies Attune NxT
Software	Attune NxT Software (v2.0+)<br>FlowJo v10 for Analysis
Cell population abundance	No sorting was performed
Gating strategy	Gating strategy is shown in Extended Data Fig. 9b. In brief, single cells were selected by FSC-H and FSC-A followed by Monocyte/ Macrophage identification using CD14-FITC-A. Histograms were generated off the Bll-A :: CD14-FITC-A positive cells for Yll-A :: CXCLlO-PE-A and YL4-A :: CD86-PE-Cy7-A

☒ Tick this box to confirm that a figure exemplifying the gating strategy is provided in the Supplementary Information.
